# Supplementary material for: Hepatic neddylation deficiency triggers fatal liver injury via inducing NF-κB-inducing kinase in mice
Source: Nat Commun. 2022 Dec 16;13:7782. doi: 10.1038/s41467-022-35525-6 (PMC9758150; doi:10.1038/s41467-022-35525-6)
Supplement: Supplementary file 1 — Supplementary information [file 41467_2022_35525_MOESM1_ESM.pdf]

SUPPLEMENTARY INFORMATION

Hepatic neddylation deficiency triggers fatal liver injury via inducing NF-κB-inducing kinase in mice

SUPPLEMENTARY METHODS

Transmission electron microscopy

For electron microscopy, freshly harvested livers were fixed overnight at 4°C in 4% PFA, 2% glutaraldehyde in 0.1 M sodium cacodylate buffer, followed by 1 h post-fixation with 2% osmium tetroxide. Upon 2% uranyl acetate treatment, samples were dehydrated with a graded series of ethanol and embedded in Epon-Araldite resin (Electron Microscopy Sciences). Sections (100 nm thickness) were cut, doubly contrasted with uranyl acetate and lead citrate, and finally imaged under a JEM 1230 transmission electron microscope (JEOL, Tokyo, Japan).

Measurement of liver triglyceride content and Oil Red O staining

Liver lipids were extracted from tissues (20-25 mg) according to a modified protocol as previously described[1]. In brief, tissues were homogenized in a solvent system consisting of chloroform/methanol/water 2:2:1.8 (v/v/v), following centrifugation at 3,500 X g for 5 min. The lower phase was transferred and evaporated by nitrogen flow. The dry extracts were dissolved in appropriate amounts of chloroform, and TG content was measured colorimetrically using the Infinity Triglycerides kit (Thermo Fisher Scientific). Data were normalized to tissue weights. To assess the hepatic steatosis, liver frozen sections (10 µm thickness) were fixed with 4% PFA and subjected to Oil red O staining using Oil Red O solution (Electron Microscopy Sciences) using standard methods[2].

Immunohistochemistry

Liver sections were deparaffinized, hydrated, and treated with 3% hydrogen peroxide to quench background peroxidase. Antigen retrieval was carried out by boiling in 10 mM citrate buffer (pH 6.0) for 10 min using a microwave. The specimens were blocked in 5% normal horse serum for

1 h at room temperature followed by overnight incubation at 4°C with primary antibodies. After washing with PBS, a biotinylated universal pan-specific antibody provided in a VECTASTAIN Elite ABC HRP kit (Vector Laboratories, Burlingame, CA) was applied for 30 min at room temperature. The specimens were subsequently exposed to avidin-biotin-peroxidase complexes for 30 min, developed using DAB Substrate Kit (Vector Laboratories), and then counterstained with hematoxylin.

Reactive oxygen species (ROS) measurement and analysis of Glutathione (GSH) and GSSG levels.

Total ROS production was measured using a method previously described with minor modifications[3]. Liver tissues were homogenized in a lysis buffer and centrifuged, then the supernatant was mixed with a dichlorofluorescein diacetate probe (DCFH-DA, Sigma-Aldrich) to a final probe concentration of 5 µM. The reaction mixture was incubated in the dark for 1 h at 37°C. Finally, the DCF fluorescence intensity was measured using a GloMax Discover microplate reader (Promega) with an excitation and emission wavelength of 485 and 530 nm respectively. Data were normalized to protein concentration as determined by the Bradford protein assay. ROS generation by mitochondria in cells was analyzed using the MitoSOX Red superoxide indicator (Invitrogen) according to the manufacturer's instructions. MitoSOX reagent was applied at a working concentration of 5 µM to incubate cells for 15 min at 37°C protected from light. Cells were counterstained with Hoechst33342 (Thermo Fisher Scientific) and imaged using a fluorescence microscope. The positive cells were counted and normalized to the total cell number. The GSH levels in hepatocytes and liver tissues were determined using a GSH/GSSG Ratio Detection Assay Kit (Fluorometric - Green) (ab138881, Abcam) or GLUTATHIONE GSH/GSSG ASSAY KIT (MAK440, Sigma-Aldrich) according to the manufacturer's instructions. Data were normalized to protein concentration or liver weights.

ATP content and cell viability assays

The intracellular ATP contents in hepatocytes were determined using an ATP Bioluminescent Assay Kit (Sigma-Aldrich) according to the manufacturer's instructions. Briefly, cells were lysed in 0.1% Triton-X100 supplemented with protease inhibitor and centrifuged. The supernatant was collected and diluted in distilled H_2O. Luminescence signals were measured using a Centro XS³ LB 960 microplate luminometer (Berthold Technologies). ATP contents were normalized to protein concentration in each sample as performed by Bradford protein assay. Hepatocyte viability was determined using a Vybrant MTT cell proliferation assay kit (Thermo Fisher Scientific) assay according to the manufacturer's instructions.

Mass spectrometry to identify neddylation sites on NIK

NIK-HA was transfected into HEK293T cells together with FLAG-NEDD8. Forty-eight hours later, denaturing cell lysates were prepared and immunoprecipitated with anti-HA magnetic beads. The beads were washed three times with cell lysis buffer and eluted with 1× loading buffer. The eluate was collected and resolved using 4-20% SDS-PAGE. Gels were coomassie blue stained using SimplyBlue Safestain (Thermo Fisher Scientific) according to Creative Proteomics (Shirley, NY). The band at or above NIK-HA was excised and subject to in-gel trypsin digestion. Peptides were extracted and subject to Nano LC-MS/MS analysis using Ultimate 3000 nano UHPLC system (Thermo Fisher Scientific, USA) coupled with mass spectrometry analysis. Raw MS files were analyzed and searched against MAP3K14 (NP_003945.2) reference sequence using Maxquant (1.6.2.6.).

Preparation of nuclear extracts from mouse liver

Nuclear extracts were prepared from freshly collected liver according to a previously published protocol with modifications [4]. Briefly, about 200 mg of liver were cut into pieces in 1xSSC buffer (150 mM NaCl, 15 mM Sodium Citrate pH 7.0) followed by vigorous shaking and centrifugation at 4,400 rpm for 2 min to remove supernatant. Pellets were resuspended in 5 ml of hypotonic buffer (10 mM Hepes pH 7.9, 1.5 mM MgCl2, 10 mM KCl, 0.2% NP40, 1 mM EDTA and 5%

sucrose) and dounced 4-5 times with 7 ml homogenizer and pestle. Liver homogenate solution was then laid on top of the 5 ml of cushion buffer (10 mM Tris pH 7.5, 15 mM NaCl, 60 mM KCl, 1 mM EDTA, and 10% sucrose) in a 15 ml tube followed by centrifugation at 5,000 rpm for 1 min, and an additional 5 min to completely remove supernatant. The nuclei pellets were then resuspended with 1 ml of TEG buffer (50 mM Tris pH 8.0, 1 mM EDTA, 15% glycerol, 500 mM NaCl), sonicated for 5 sec on ice slurry, and incubated on ice for 30 min. Nuclear extracts were then collected after ultracentrifugation at 46,000 rpm for 30 min. All procedures were performed at 4 °C with protease and phosphatase inhibitors freshly added to the buffers.

SUPPLEMENTARY TABLES

Supplementary Table 1. Primary and secondary antibodies used for immunoblotting, immunoprecipitation, and immunostaining

Antibody	Supplier (Catalog No.)	Dilution
ACTB (clone C4)	Sigma-Millipore (MAB1501)	1:5000
Albumin	Novus Biologicals (NB600-41532) (IF)	1:100
Catalase	Genetex (GTX110704)	1:1000
C/EBPα	Santa Cruz (sc-61)	1:500
CK19	Abbomax (602-670) (IF)	1:500
Pan-Cytokeratin (clone K4.62)	Sigma Aldrich (C7159) (IF)	1:20
Cleaved caspase-3	Cell Signaling Technology (9661)	1:1000
Cleaved caspase-8	Cell Signaling Technology (9429)	1:1000
CTNB1 (clone D10A8)	Cell Signaling Technology (8480)	1:1000
CUL1	Santa Cruz (sc-1384)	1:1000
CUL2 (clone EPR3104(2))	Abcam (ab166917)	1:1000
CUL3	Cell Signaling Technology (2759)	1:1000
CUL4a	Novus Biologicals (NB100-2267)	1:1000
Desmin (clone DE-U-10)	Sigma Aldrich (D1033) (IF)	1:40
FLAG (clone M2)	Sigma Aldrich (SLBD6976)	1:1000
F4/80 (clone Cl:A3-1)	Bio-Rad (MCA497GA) (IF)	1:50
GAPDH (clone 1E6D9)	Proteintech (60004-1-IG)	1:20000
GFP	Abcam (ab290)	1:1000
HA (clone C29F4)	Cell Signaling Technology (3724)	1:1000
Anti-HA magnetic beads	ThermoFisher Scientific (PI88836)	
HNF4α (clone F.674.9)	ThermoFisher Scientific (MA5-14891)	1:500
HNF4α (clone K9218)	ThermoFisher Scientific (MA1-199)	1:1000
IκBα (clone L35A5)	Cell Signaling Technology (4814)	1:1000
p-IκBα (clone 14D4)	Cell Signaling Technology (2859)	1:1000
Ki-67 (clone D3B5)	Cell Signaling Technology (9129) (IF)	1:400
LMNB2	Proteintech (10895-1-AP)	1:2000
MLKL	Abcam (ab172868)	1:1000
MYC (clone 9B11)	Cell signaling technology (2276)	1:1000
Anti-MYC magnetic beads	ThermoFisher Scientific (PI) (IP)	
NAE1 (clone D9I4Z)	Cell Signaling Technology (14321)	1:1000
NEDD8 (clone 19E3)	Cell Signaling Technology (2754)	1:1000
NEDD8 (clone 19E3)	Cell Signaling Technology (2754) (IF)	1:300
p-NF-κB2 p100(Ser866/870)	Cell Signaling Technology (4810)	1:1000
NF-κB2 p100/p52	Cell Signaling Technology (4882)	1:1000
NIK	Cell Signaling Technology (4994)	1:1000
NIK (clone A-12)	Santa Cruz Biotechnology (sc-8417)	
NOX2 (clone 53)	BD Biosciences (611414)	1:1000
NRF2 (clone D1Z9C)	Cell Signaling Technology (4994)	1:1000
P53 (clone D2H9O)	Cell Signaling Technology (32532)	1:500
P65 (clone D14E12)	Cell Signaling Technology (8242)	1:1000
RBX2	Proteintech (11905-1-AP)	1:1000
p-RIPK3(T231+S232) (clone EPR19403-52)	Abcam (ab201912)	1:5000
RIPK3	Novus Biologicals (NBP1-77299)	1:2000

RIPK3	Novus Biologicals (NBP1-77299)	1:200
SOD1	Proteintech (10269-1-AP)	1:1000
SOD2	Proteintech (24127-1-AP)	1:5000
UBC12 (clone D13D7)	Cell Signaling Technology (5641) (IB)	1:1000
UBC12 (clone D-4)	Santa Cruz (sc-390064) (IP)	
Ubiquitin (clone P4D1)	Cell Signaling Technology (3936)	1:1000
Xanthine oxidase (clone A-3)	Santa Cruz (sc-398548)	1:500
YAP (clone D8H1X)	Cell Signaling Technology (14074)	1:1000
Alexa Fluor 488-conjugated goat anti-Rabbit IgG (H+L)	ThermoFisher (A11034)	1:500
Alexa Fluor 594-conjugated F(ab')2-goat anti-Rabbit IgG (H+L)	ThermoFisher (A11072)	1:500
Alexa Fluor 594-conjugated goat anti-mouse IgG (H+L)	ThermoFisher (A11032)	1:500
Goat anti-Rat IgG (H+L) Cross Adsorbed Secondary Antibody, Alexa Fluor 647	ThermoFisher (A21247)	1:500
Alexa Fluor Plus 594-conjugated donkey anti-Goat IgG (H+L)	ThermoFisher (A32758)	1:500
Alexa Fluor 488-conjugated donkey anti-rabbit IgG (H+L)	ThermoFisher (A21206)	1:500
HRP-conjugated goat anti-rabbit IgG (H+L)	Bio-rad, 1706515	1:300
HRP-conjugated goat anti-mouse IgG (H+L)	Bio-rad, 1706516	1:300

Supplementary Table 2. Primer sequences used for qRT-PCR

Gene	Species	Forward Primer Sequence	Reverse Primer Sequence
Alb	mouse	GCAGATGACAGGGCGGAACTTG	CAGCAGCAATGGCAGGCAGAT
Acta2	mouse	CACTGAACCCTAAGGCCAAC	CATCTCCAGAGTCCAGCACA
Adgre1	mouse	CTTTGGCTATGGGCTTCCAGTC	GCAAGGAGGACAGAGTTTATCGTG
AMOTL2	human	GCTTCAATGAGGGTCTGCTC	GATCACTGCATCCTTCTCCA
Cebpa	mouse	GAACAGCAACGAGTACCGGGTA	GCCATGGCCTTGACCAAGGAG
CEBPa	human	GTGGACAAGAACAGCAACGA	GGTCAGCTCCAGCACCTT
Ccl2	mouse	CAGCAAGATGATCCCAATGA	TGTCTGGACCCATTCCTTCT
Cd68	mouse	CCAATTCAGGGTGGAAGAAA	GACTGTACTCGGGCTCTGATG
Cd34	mouse	GGGTAGCTCTCTGCCTGATG	TGGCTGGTGTGGTCTTACTG
Col1a1	mouse	AAGACGGACTCAACGGTCTC	CTGAGGCAGGAAGCTGAAGT
Col1a2	mouse	GTCCTAGTCGATGGCTGCTC	GTCAGCACCACCAATGTCC
Col3a1	mouse	ACGTGGTAGTCCTGGTGGTC	GTCCAGCTATTCCAGGGTTG
Col4a1	mouse	TTTGACATGCGACTCAAAGG	CGATGTCTCCACGACTACCA
Col6a1	mouse	TCTGACACTCAACGGGACAC	TGGCAGGAAATGACATTGAG
Ccn2	mouse	CTTCCCGAGAAGGGTCAAG	GGCCAAATGTGTCTTCCAGT
Cyp1a2	mouse	CTCACTGAATGGCTTCCACA	CCGAAGAGCATCACCTTCTC
Cyp2a4	mouse	GGTCACCAAGGACACCAAGT	GGAAGTGCTTTGGGTTGAAG
Cxcl14	mouse	AAGCTGGAAATGAAGCCAAA	CCAGGCATTGTACCACTTGA
Esrp2	mouse	ACAGAGGAGATGAGCCGTGT	CTGGAAGGTGGCGTAGGTAG
Fbp1	mouse	TCAACTGCTTCATGCTGGAC	AGTCCTTGGCATAACCCTCA
Gclc	mouse	GGATTCACACTGCCAGAACA	TGGCACATTGATGACAACCT
Gclm	mouse	CAAGTTAACCTGGCCTCCTG	TCAATGTCAGGGATGCTTTCT
Gss	mouse	CTTGCTGCTCCTAGCCACTT	AGGCTCTCTCCTCACTGTCCT
Gsr	mouse	CAGATGTTGACTGCCTGCTC	GACGTCTCCCACAGCATAGAC
Gls2	mouse	CCGTGGTGAACCTGCTATTT	GTGCGGGAATCATAGTCCTT
Hnf4a	mouse	GCATGGATATGGCCGACTACA	ACCTTCAGATGGGGACGTGT
HNF4a	human	CGTGGTGGACAAAGACAAGA	GCTTGACCTTCGAGTGCTG
Hmgcs2	mouse	CCTACCGCAAGAAGATCCAG	CACTGCTGGATGACAGGAAG
Itgax	mouse	TGATGAGCCAGCTTCAGAGA	GCTGCCTTACAGAACCCAAC
Krt19	mouse	CGGACCCTCCCGAGATTACA	TGGAGTTGTCAATGGTGGCA
KRT19	human	GACATGCGAAGCCAATATGA	TCAGTAACCTCGGACCTGCT
Krt18	mouse	CGAGGCACTCAAGGAAGAAC	GCATCCACTTCCACAGTCAA
Lgals3	mouse	CCTATGACCTGCCCTTGC	CCCAGTTGGCTGATTTCC
Mgst3	mouse	TTGTGCTTCTCACTGGTGCT	AACATATGCCCGTTCTCAGG
Map3k14	mouse	TGACATCCCGAGCTACTTCA	AGCAAACAGGCTGTCCATCT
MAP3K14	human	TGTGGGAACCCTTACCTCTG	CGAGAGAATTTGCTCCTGCT
Opn	mouse	CTGACCCATCTCAGAAGCAGAATCT	TCCATGTGGTCATGGCTTTCATTGG
OPN	human	GCCGAGGTGATAGTGTGGTT	TGAGGTGATGTCCTCGTCTG
Pkm	mouse	CGATCTGTGGAGATGCTGAA	TTCACGGACATTCTTGATGG
PKM	human	TGTCTGGAGAAACAGCCAAA	CCATGAGGTCTGTGGAGTGA
Pck1	mouse	CCATCACCTCCTGGAAGAAC	ACGGCCACCAAAGATGATAC
PCK1	human	ACATCTGTGACGGCTCTGAG	GGATGGGCACTGTGTCTCTT
Ppara	mouse	CCACGAAGCCTACCTGAAGA	ACTGGCAGCAGTGGAAGAAT
Pgc1a	mouse	CCCTGCCATTGTTAAGACC	TGCTGCTGTTCCTGTTTTC
Srebf1	mouse	AGGCCCGGGAAGTCACTGT	GGAGCCATGGATTGCACATT
Sox9	mouse	CAGACCAGTACCCGCATCT	CTCCTCCACGAAGGGTCTC
Tnf	mouse	TGCCTATGTCTCAGCCTCTTC	GGAGGCCATTTGGGAACT

Supplementary Table 3. Summary of plasmids including names, features, and sources.

Plasmids name	Features	Sources
pCMV4-HA	pCMV4 empty vector with CMV promoter	Addgene#27553[5]
pCMV4-NIK-HA	Human NIK with C-terminal 1x HA tag	Addgene#27554[5]
pcDNA3.1 (+)-NIK-HA	Human NIK with C-terminal 1x HA tag (MAP3K14_OHu27997C)	GenScript#SC1200
pcDNA3.1 (+)- NIK-K385R-HA	Human NIK K385R with C-terminal 1xHA tag	GenScript#SC1200M
pcDNA3.1 (+)-NIK-K387R-HA	Human NIK K387R with C-terminal 1xHA tag	GenScript#SC1200M
pcDNA3.1 (+)- NIK-K607R-HA	Human NIK K607R with C-terminal 1xHA tag	GenScript#SC1200M
pcDNA3.1 (+)- NIK-K650R-HA	Human NIK K650R with C-terminal 1xHA tag	GenScript#SC1200M
pcDNA3.1 (+)- NIK-K4R-HA	Human NIK K385R, K387R, K607R, K650R with C-terminal 1xHA tag	GenScript#SC1200M
pcDNA3.1 (+)- NIK-K49R-HA	Human NIK K49R, with C-terminal 1xHA tag	GenScript#SC1626
pcDNA3.1 (+)- NIK-K29R-HA	Human NIK K29R with C-terminal 1xHA tag	GenScript#SC1625
pcDNA3.1 (+)- NIK-K20R-HA	Human NIK K20R with C-terminal 1xHA tag	GenScript#SC1625
pcDNA3.1 (+)- NIK-Myc	Human NIK with C-terminal 1xMyc tag	Subcloned from SC1200
pShuttle-CMV-SF-NEDD8	Human NEDD8 with N-terminal streptavidin & FLAG-tag	Dr. Huabo Su Lab
pShuttle-CMV-SF-NEDD8ΔGG	Human NEDD8 mutant with N-terminal streptavidin & FLAG-tag	Dr. Huabo Su Lab
pcDNA3.1 (+)-UBB	Human UBB (NM_018955.4)	GenScript OHu04823D
pRK5-HA-Ubiquitin-WT	Human N-terminal HA-tagged ubiquitin	Addgene#17608[6]
PFLAG-CMV-SENP8WT	Human wild-type SENP8 with N-terminal FLAG tag	Addgene#18066[7]
PFLAG-CMV-SENP8Mut	Human SENP8 C153A with N-terminal FLAG tag	Addgene#18717[7]
pLentiCRISPR V2	All-in-one lentiviral vector expressing Cas9 nuclease with U6 promoter	Addgene# 52961[8]
pLentiCRISPR V2 human NAE1 sgRNA1	sgRNA1: TCAAGGAGCAGAAGTACGAC against human NAE1 exon 1	Genscript#SC1805
pLentiCRISPR V2 human NAE1 sgRNA3	sgRNA3: AGGCGCGGCCATGGCGCAGC against human NAE1 exon1 ATG start site	GenScript#SC1805
pLentiCRISPR V2 human CUL2 sgRNA1	sgRNA1: TTTGACGACAATAAAAGCCG against human CUL2 exon 2	Genscript#SC1805
pLentiCRISPR V2 human CUL3 sgRNA5	sgRNA5: GACCTAAAATCATTAACATC against human CUL3 exon 5	Genscript#SC1678
pLentiCRISPR V2 mouse Cul1 sgRNA1	sgRNA1: GTTCGCCGTGAATGTGATGA against mouse Cul1 exon 3	GenScript#SC1805
pLentiCRISPR V2 mouse Cul1 sgRNA2	sgRNA2: TTCGTCCTTCATCACATTCA against mouse Cul1 exon 3	GenScript#SC1805
pLentiCRISPR V2 mouse Cul4a sgRNA4	sgRNA4: GCTCTACAAGCAGCTGCGCC against mouse Cul4a exon 3	GenScript#SC1805
pLentiCRISPR V2 human RBX2 (gene RNF7) sgRNA2	sgRNA2: CCACCGCGTTCCACTTCTTG against human RBX2 exon 1	GenScript#SC1805
pMD2.G VSV G	VSV-G envelope expressing plasmid	Addgene# 12259
psPAX2	2nd generation lentiviral packaging plasmid	Addgene# 12260

Supplementary Table 4. Summary of DsiRNA and siRNA sequences.

Gene name	Sequences	Sources
Human SENP8	Seq 1: rUrCrArUrUrArCrCrArCrArCrUrUrGrCrUrArArArArArGTA<br>Seq 2: rUrArCrUrUrUrUrUrArGrCrArArGrUrGrUrGrGrUrArArUrGrArGrA	IDT DsiRNA#1: hs.Ri.SENP8.13.2
Human SENP8	Seq 1: rArGrArCrUrUrUrUrCrUrUrArUrUrGrGrUrArUrArUrArATT<br>Seq 2: rArArUrUrArUrArUrArCrCrArArUrArArGrArArArArGrUrCrUrUrA	IDT DsiRNA#2: hs.Ri.SENP8.13.3
Human UBC12	Seq 1: rGrArCrGrUrGrUrGrArUrArUrCrArGrCrUrUrCrUrCrArGAT<br>Seq 2: rArUrCrUrGrArGrArArGrCrUrGrArUrArUrCrArCrArCrGrUrCrUrU	IDT DsiRNA: CD.Ri.390331.13.1
Human NEDD8	GACAGCAGCTGATTACAAG	Sigma siRNA #1
Human NEDD8	TATATGATGCCTCATTATG	Sigma siRNA #2

Supplementary Figures and Legends

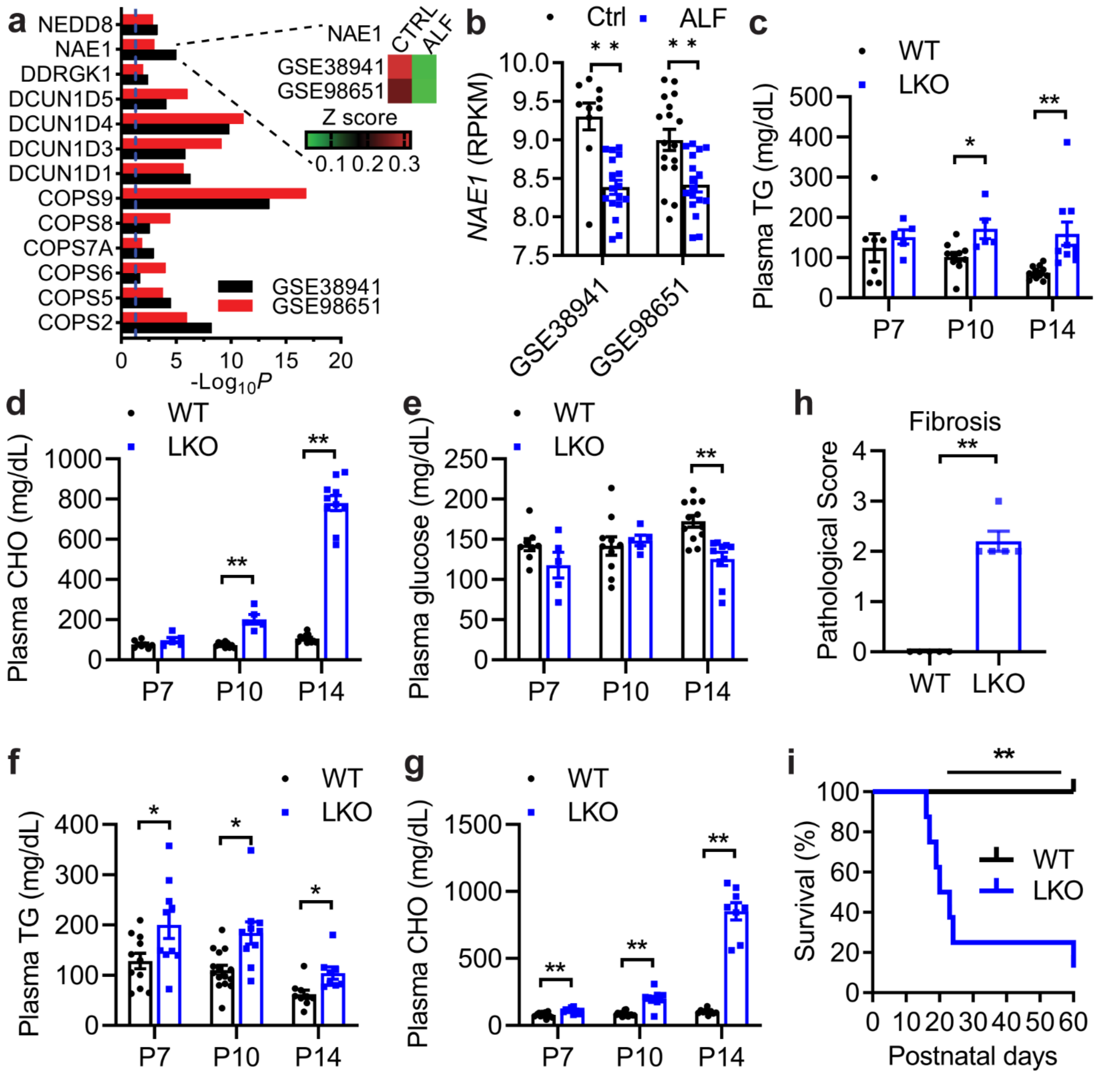

Fig. S1. Embryonic onset, hepatocyte-specific deletion of NAE1 causes rapid progressive liver injury and premature death.

a Bar graph showing differentially expressed genes related to neddylation (-Log$_{10}$P>1.3, dashed line) in patients with HBV-associated acute liver failure (ALF) relative to healthy control (Ctrl) individuals from two GSE datasets (GSE38941 in black and GSE98651 in red) obtained from

the NCBI GEO database. Insert indicates the heatmap of *NAE1* expression in both datasets. Two-sided unpaired t-test. **b** Distribution of human *NAE1* expression (RPKM) in Ctrl (black) and ALF (blue) groups from two GSE datasets, respectively. **c** Plasma triglyceride (TG), **d** cholesterol (CHO) and **e** glucose levels at postnatal (P)7 (WT: n = 7; LKO: n = 5), P10 (WT: n = 10; LKO: n = 5) and P14 (WT: n = 12; LKO: n = 10) in male WT and LKO mice. **f** Plasma TG and **g** CHO levels at P7 (WT: n = 11; LKO: n = 10), P10 (WT: n = 15; LKO: n = 10) and P14 (WT: n = 9; LKO: n = 8) in female WT and LKO mice. Multiple unpaired t-tests with the Holm-Sidak method in (**b-g**). *P < 0.05, **P < 0.005. **h** Pathological scores of liver fibrosis based on Sirius red staining at P14 in female WT and LKO livers (n = 5 per group). Unpaired t-tests, two-tailed. **P < 0.0001. **i** Survival curves of female WT (n = 7) and LKO (n = 8) mice during the monitoring period as indicated. Log-rank (Mantel-Cox) test. **P = 0.0009. In (**c-i**), WT (black), LKO (blue). All quantitative data were presented as mean ± SEM. Quantitative source data are provided as a Source Data file.

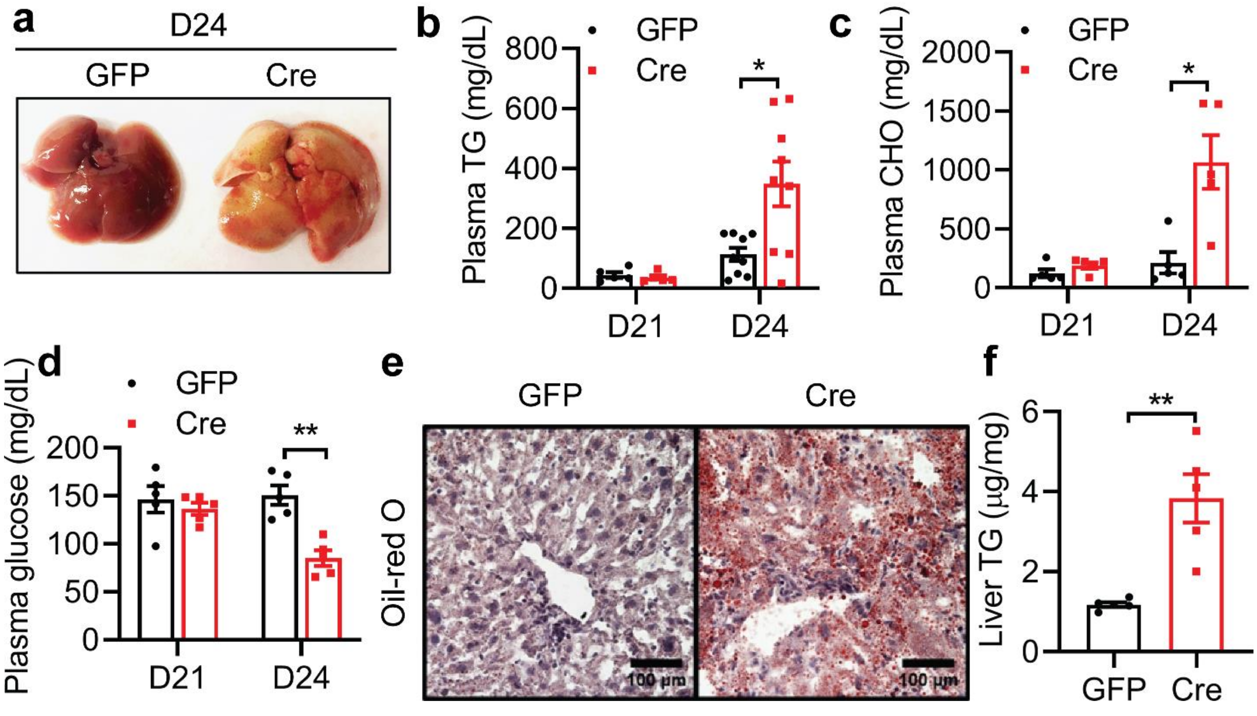

Fig. S2. **Adult-onset, hepatocyte-specific deletion of NAE1 causes rapid progressive liver injury and premature death.**

a Gross morphology of livers from D24 GFP and Cre mice. **b** Plasma TG, **c** cholesterol (CHO) and **d** glucose levels in male *Nae1*$^{f/f}$ mice at D21 (*n* = 5 per group) and D24 (GFP: *n* = 5; Cre: *n* = 5-9) post-injection of AAV8-TBG-Cre (AAV-Cre, a.k.a. Cre) or AAV8-TBG-eGFP (AAV-GFP, a.k.a. GFP). In (**b-d**)**,** Multiple t-tests with the Holm-Sidak method. *$P < 0.05$, **$P < 0.005$. **e** Representative Oil Red O staining and **f** enzymatic quantification of liver TG contents as normalized to tissue weights in male GFP and Cre mice at D24 post virus injections (*n* = 5 per group). Scale bar: 100 µm. Unpaired t-tests, two-tailed. **$P = 0.0024$. GFP: black; Cre: red. All quantitative data were presented as mean ± SEM. Quantitative source data are provided as a Source Data file.

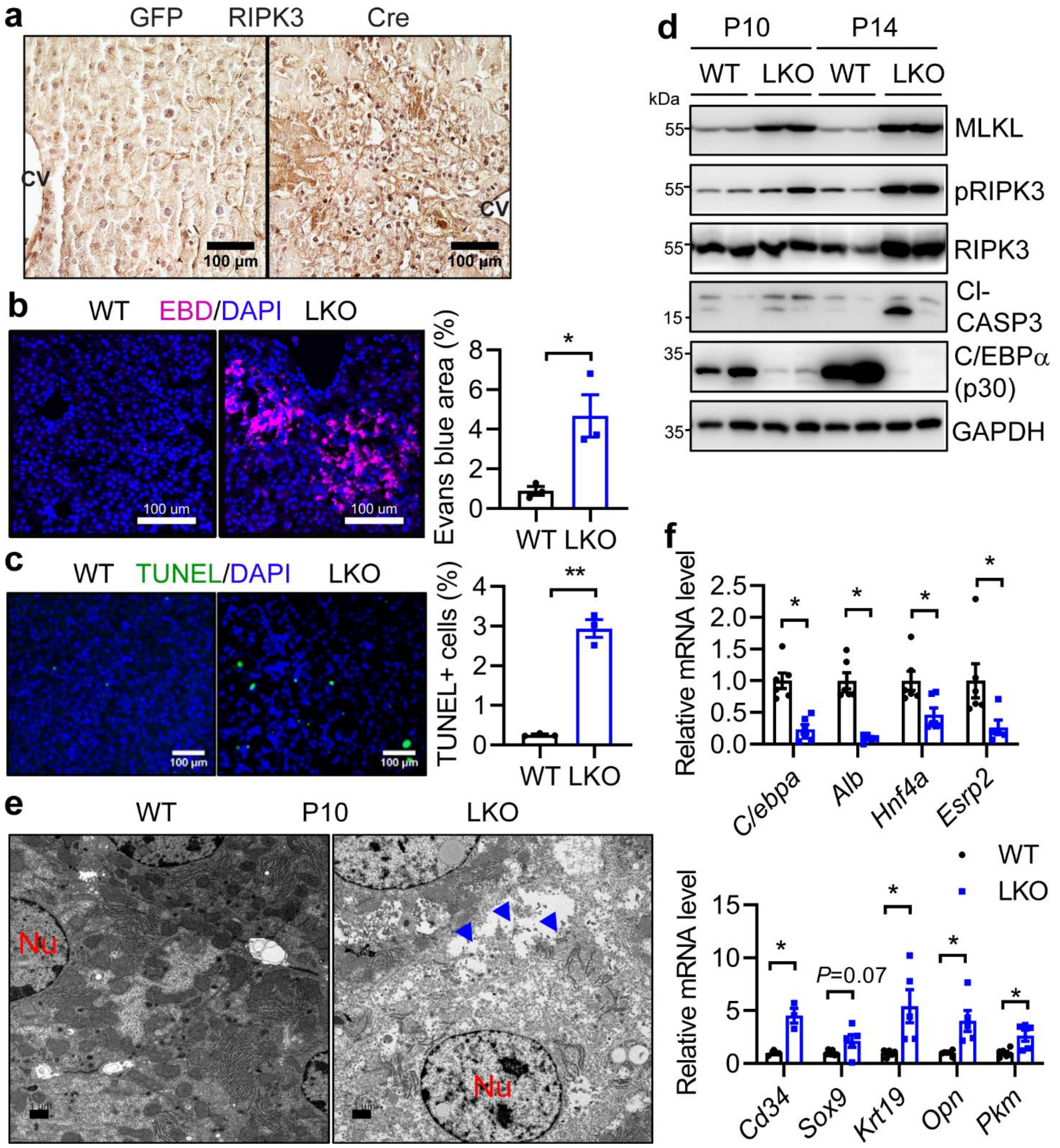

Fig. S3. Hepatocyte cell death and aberrant fetal reprogramming in mice with adult-onset and embryonic-onset hepatocyte-specific deletion of NAE1.

a Representative immunohistochemistry of RIPK3 in livers from D24 AAV-GFP (GFP) and AAV-Cre (Cre) mice (*n* = 3 per group). Scale bar: 100 µm. CV: central vein. **b** Evans blue dye (EBD) staining and quantification of total EBD^+ areas (**P* = 0.0255); **c** TUNEL labeling and quantification

of TUNEL^{+} cells (**P = 0.0003) in livers of P14 *Nae1*$^{f/f}$ (WT) and *Nae1*$^{f/f}$; *Alb-Cre*+ (LKO) mice (*n* = 3 per group). Scale bar: 100 µm. Two-tailed unpaired t-tests in (**b-c**). **d** Representative Western blot analysis in P10 and P14 liver extracts (*n* = 4 per group). **e** Representative TEM micrographs of P10 WT and LKO livers from four biologically independent samples. Blue arrowheads indicate ruptured membranes representing cells undergoing necroptosis. Nu, nucleus. Scale bar: 1 µm. **f** Relative mRNA levels of mature hepatocyte markers and BEC/progenitor-specific markers in livers of P14 male WT and LKO mice (*n* = 6 per group). Multiple unpaired t-tests with the Holm-Sidak method. *P < 0.05, **P < 0.005. WT (black); LKO (blue). All quantitative data were presented as mean ± SEM. Quantitative source data are provided as a Source Data file. Uncropped gels are provided in the Supplementary file.

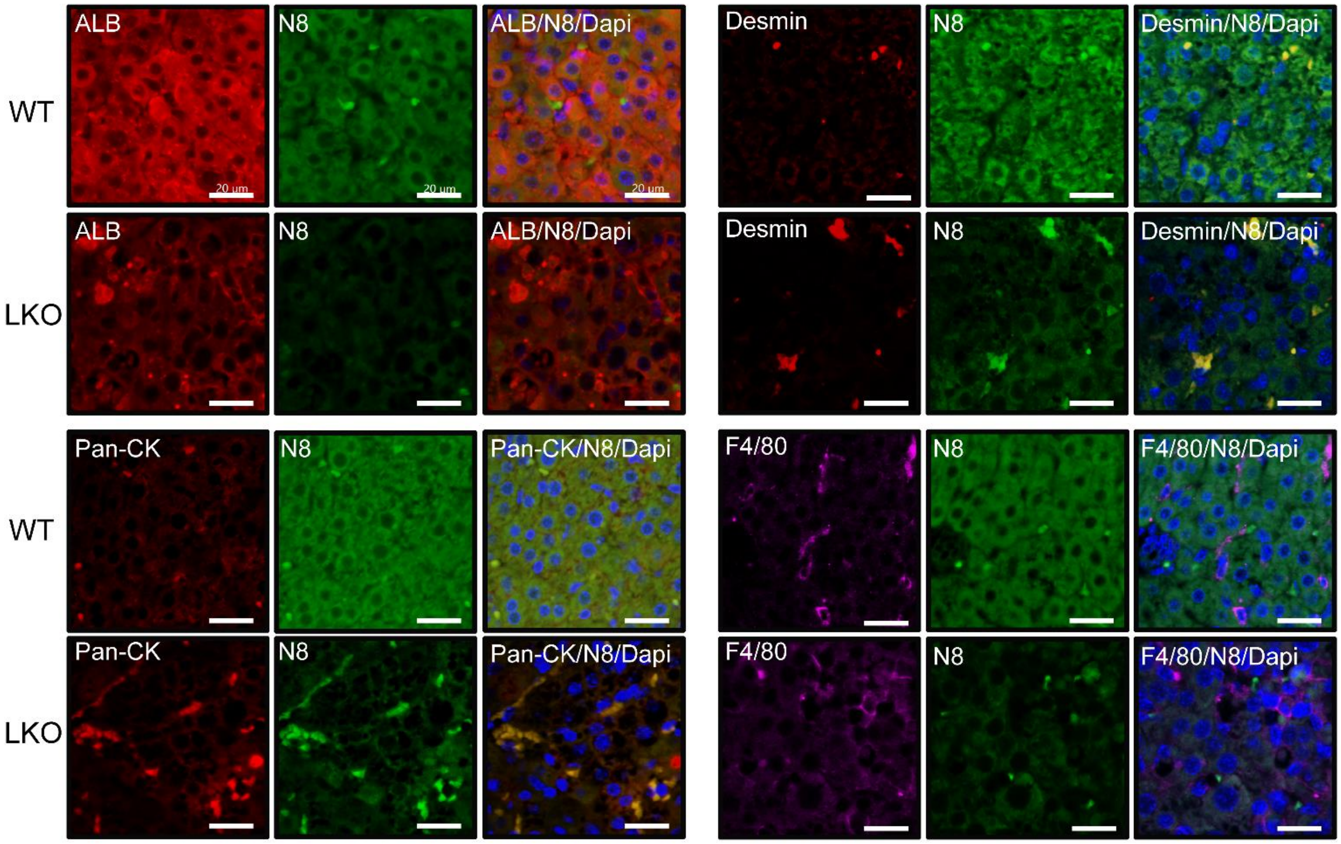

Fig. S4. Aberrant fetal reprogramming and neddylation patterns in mouse livers with embryonic-onset deletion of NAE1.

Representative individual and merged immunofluorescence for Albumin (ALB), a hepatocyte marker; Desmin, a hepatic stellate cell marker; Cytokeratin (Pan-CK), BEC/progenitor cell marker, F4/80, secreted by activated Kupffer cells, with NEDD8 (N8) in male P14 *Nae1*$^{f/f}$ (WT) and *Nae1*$^{f/f}$; *Alb-Cre+* (LKO) mice liver, respectively. Scale bar: 20 µm. *n* = 3 biologically independent samples.

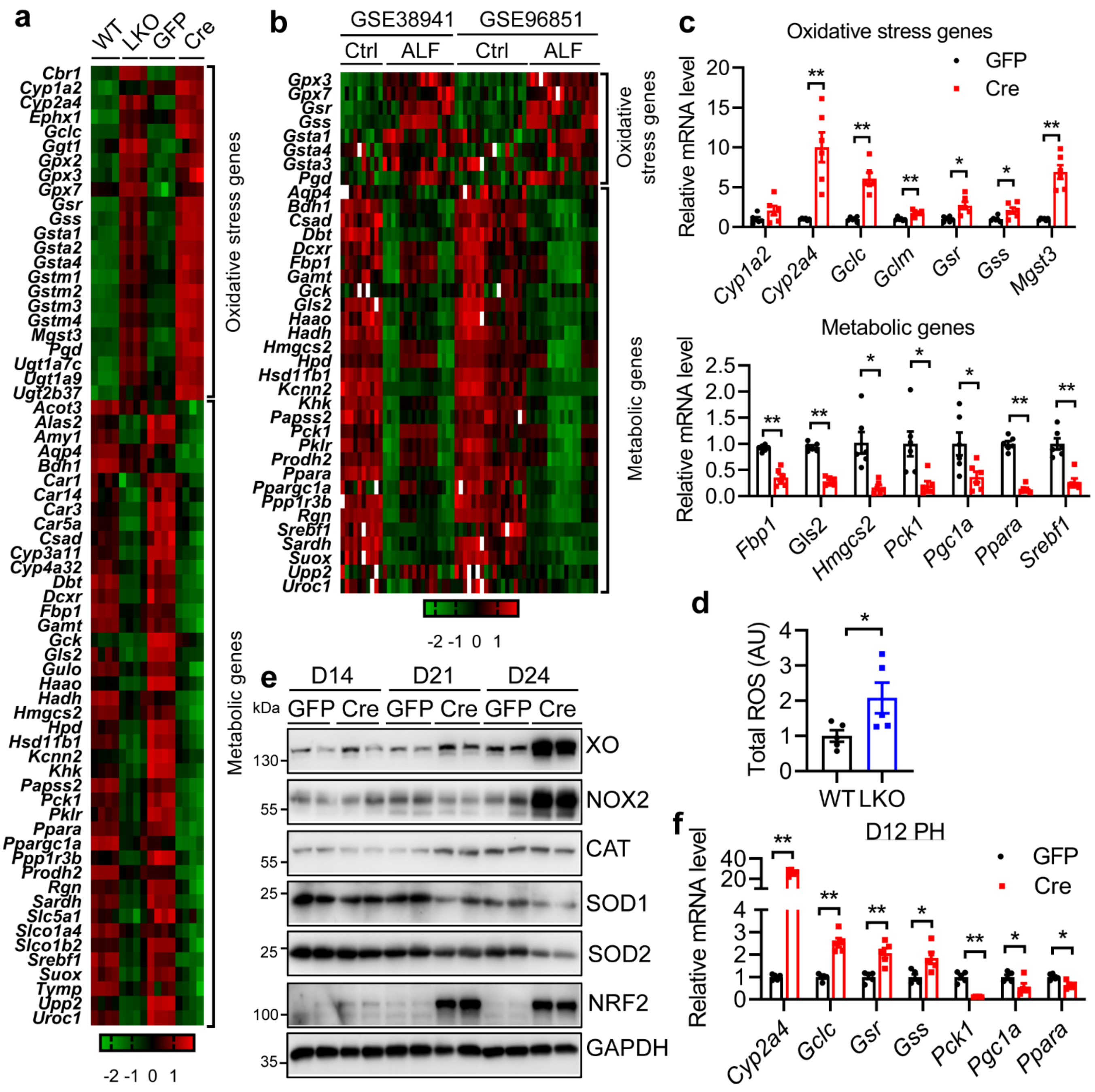

Fig. S5. Hepatocyte-specific neddylation deficiency causes oxidative stress and mitochondrial dysfunction.

a Heatmap presentation of DEGs related to oxidative stress and metabolism in livers from male LKO and Cre mice as compared to WT and GFP counterparts, respectively. **b** Heatmap presentation of DEGs related to oxidative stress and metabolism in human livers from Ctrl and

ALF patients obtained from GSE3899941 and GSE96851, respectively. **c** qRT-PCR confirmation of mRNA changes of major DEGs in oxidative stress and metabolism pathways in livers of D21 GFP (black) and Cre (red) mice (n = 6 per group). Multiple unpaired t-tests with the Holm-Sidak method. *P < 0.05, **P < 0.005. **d** ROS levels were measured by incubating liver homogenates with a DCFH-DA probe and expressed as AU from male P10 WT (black) and LKO (blue) mice (n = 5 per group). Unpaired t-tests, two-tailed, *P = 0.0487. **e** Representative Western blot in liver extracts of D14, D21, and D24 GFP and Cre mice (n = 4 per group). **f** Relative mRNA levels of indicated genes in primary hepatocytes (PH) isolated from livers of D12 GFP (black) and Cre (red) mice (n = 5 per group). Three biologically independent experiments. Multiple t-tests with the Holm-Sidak method. *P < 0.05, **P < 0.005. All quantitative data were presented as mean ± SEM. Quantitative source data are provided as a Source Data file. Uncropped gels are in the Supplementary file.

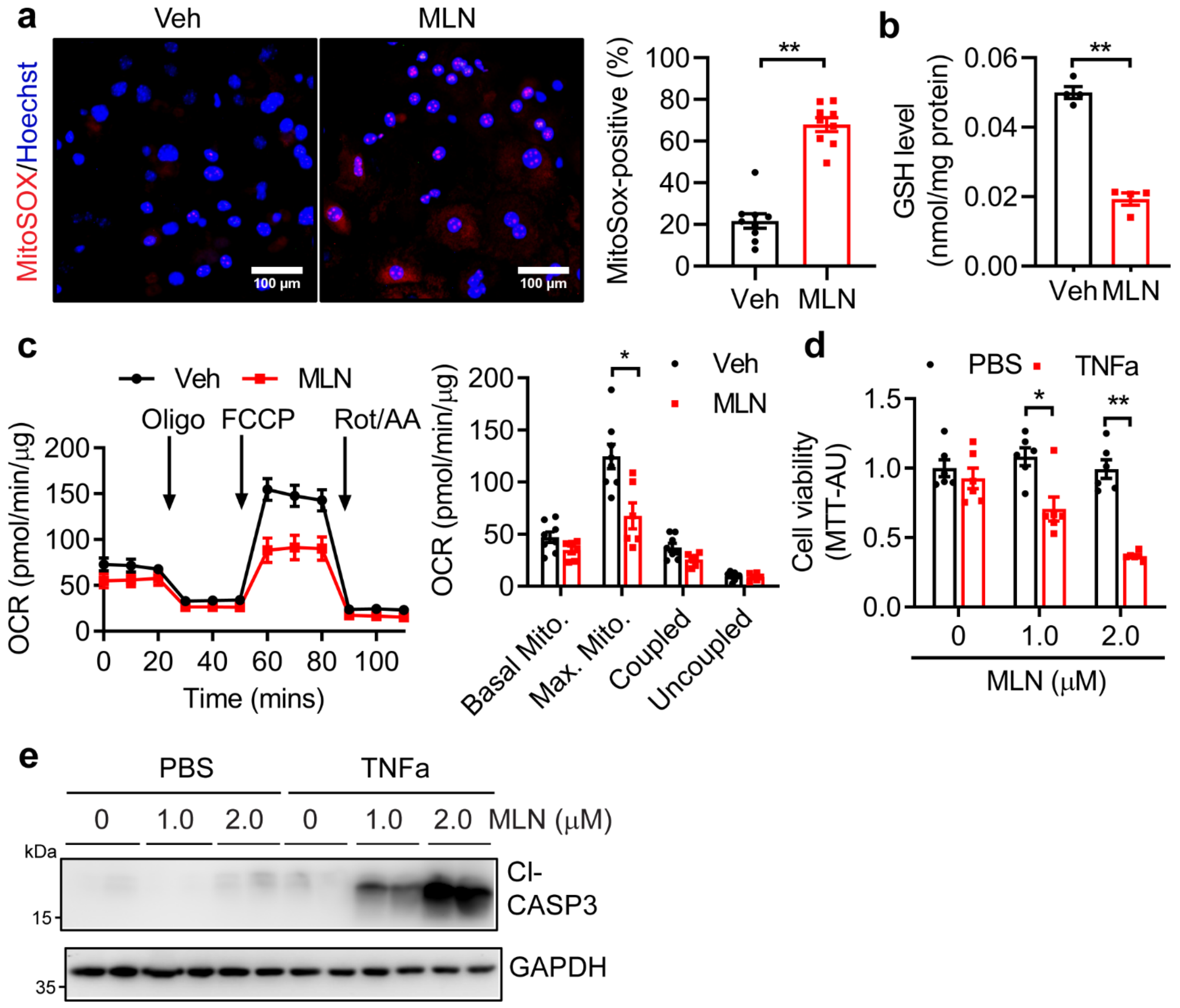

Fig. S6. Neddylation inhibition induces oxidative stress and mitochondrial dysfunction *in vitro*.

a-e All experiments were performed in primary hepatocytes isolated from male C57BL/6J mice treated with vehicle (Veh) or 2.0 µM MLN for 48 h. **a** Representative MitoSOX staining and quantification of MitoSOX-positive cells. Scale bar: 100 µm. **b** Total GSH levels were measured and normalized to protein amounts (n = 4). **a-b**: Unpaired t-tests, two-tailed. **c** Oxygen consumption rate (OCR) was detected using a Seahorse XF Cell Mito Stress Test. Basal,

maximal (Max.), coupled, and uncoupled mitochondrial (Mito.) respirations were shown in the right panel. **d** Cell viability was determined by MTT assays and expressed as arbitrary units (AU) (*n* = 6 per group). Multiple unpaired t-tests with the Holm-Sidak method in **c-d**. **e** Representative Western blot of indicated proteins in primary hepatocytes treated with Veh or indicated concentrations of MLN for 48 h with or without 20 ng/mL TNFα in the last 24 h Veh (black), MLN (red). Representative data from three biologically independent experiments were shown for **a-e**. $^*P < 0.05$, $^{**}P < 0.005$. All quantitative data were presented as mean ± SEM. Quantitative source data are provided as a Source Data file. Uncropped gels are provided in the Supplementary file.

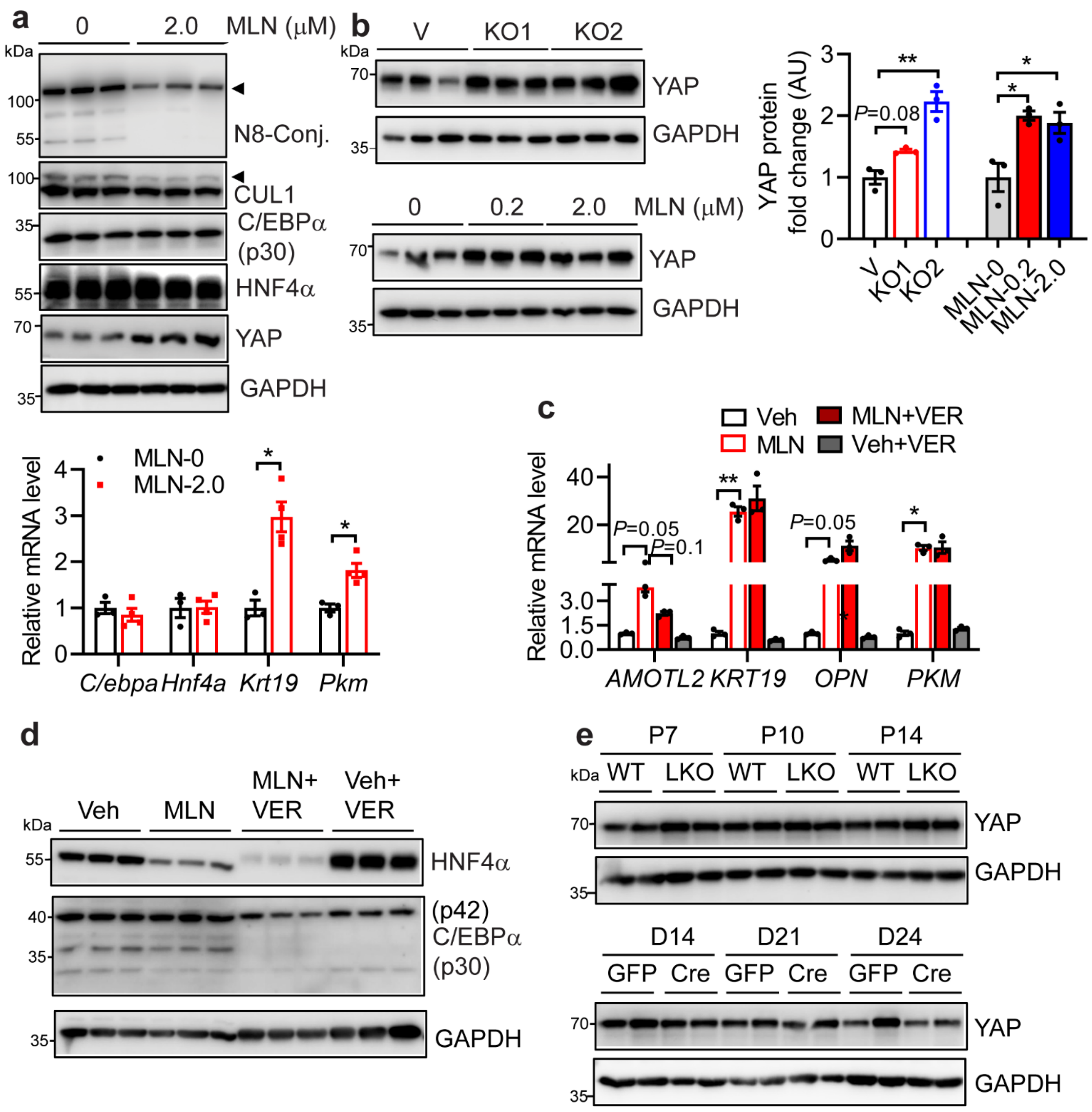

Fig. S7. Neddylation deficiency-induced hepatocyte fetal reprogramming is independent of YAP activation.

a Representative Western blot and relative mRNA levels of indicated genes in primary hepatocytes (PH) isolated from male C57BL/6 mice and treated with 0 (*n* = 3 per group) and 2.0 μM MLN (*n* = 4 per group) for 96 h. Multiple unpaired t-tests with the Holm-Sidak method. *P <

0.05. MLN-0: black; MLN-2.0: red. **b** Representative Western blot and quantification of YAP in NAE1 KO1 and KO2 HepG2 cells and MLN-treated HepG2 cells at indicated concentrations for 48 h (*n* = 3 per group). One-way ANOVA followed by Tukey's multiple comparisons test. *P < 0.05. **c** Relative mRNA levels of indicated genes (*n* = 3 per group), and **d** Western blot of indicated proteins in HepG2 cells treated with vehicle (Veh) or 2.0 µM MLN in the presence or absence of 10 µM Verteporfin (VER) for 48 h (*n* = 3 per group). Two-way ANOVA followed by Tukey's multiple comparisons test. *P < 0.05, **P < 0.005. Two biologically independent experiments for **a-d**. **e** Western blot of YAP in livers of male *Nae1*$^{f/f}$ (WT) and *Nae1*$^{f/f}$; *Alb-Cre*+ (LKO) or AAV-GFP (GFP) and AAV-Cre (Cre) mice at indicated ages or days post virus injections (*n* = 4 per group). All quantitative data were presented as mean ± SEM. Quantitative source data are provided as a Source Data file. Uncropped gels are provided in the Supplementary file.

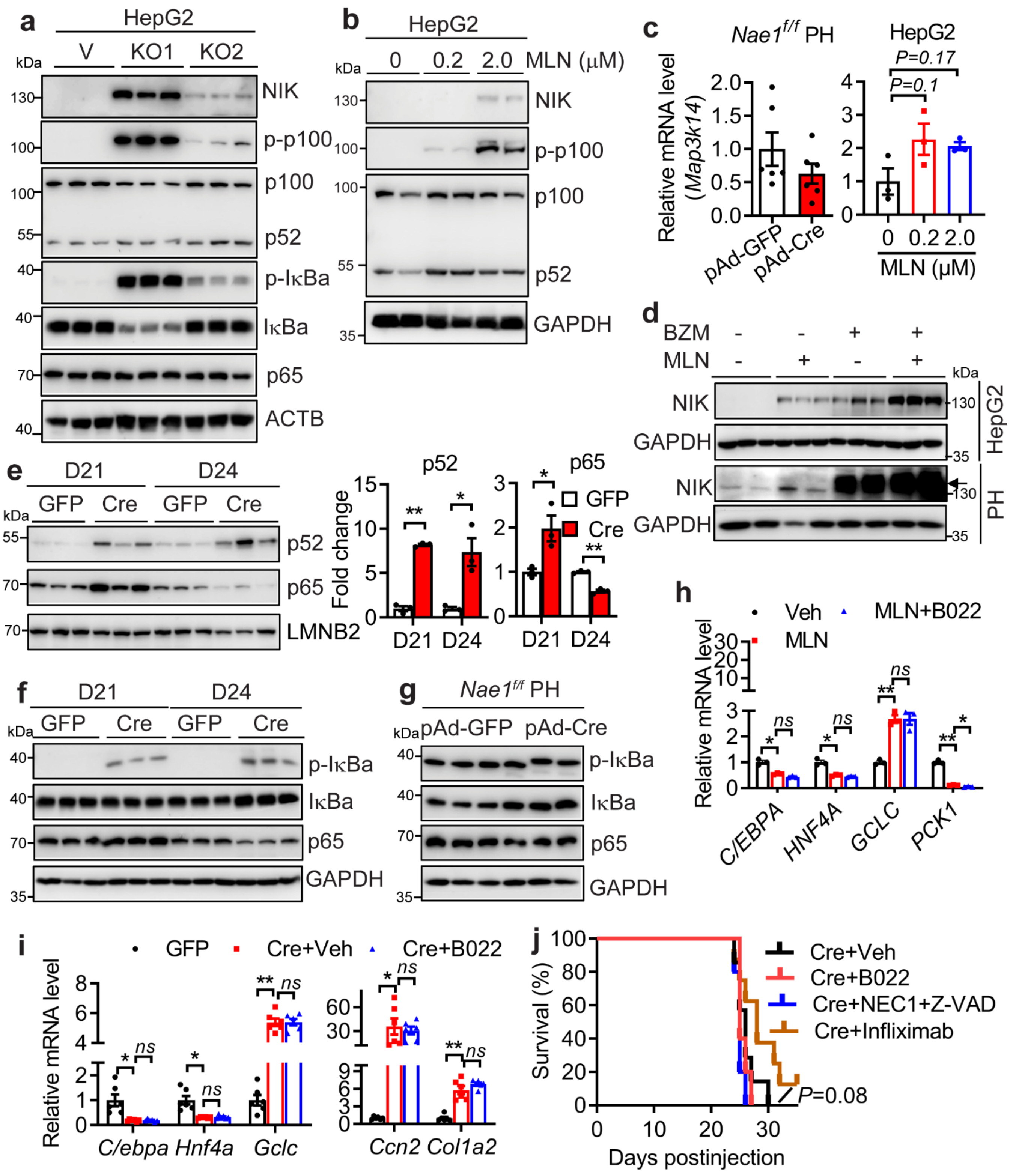

a
HepG2
V
KO1
KO2
NIK
p-p100
p100
p52
p-IκBa
IκBa
p65
ACTB
b
HepG2
0
0.2
2.0
MLN (μM)
NIK
p-p100
p100
p52
GAPDH
c
Nae1f/f PH
HepG2
Relative mRNA level (Map3k14)
pAd-GFP
pAd-Cre
P=0.17
P=0.1
MLN (μM)
d
BZM
MLN
NIK
GAPDH
HepG2
PH
e
D21
D24
GFP
Cre
p52
p65
LMNB2
Fold change
p52
p65
GFP
Cre
f
D21
D24
GFP
Cre
p-IκBa
IκBa
p65
GAPDH
g
Nae1f/f PH
pAd-GFP
pAd-Cre
p-IκBa
IκBa
p65
GAPDH
h
Veh
MLN+B022
MLN
Relative mRNA level
C/EBPA
HNF4A
GCLC
PCK1
i
GFP
Cre+Veh
Cre+B022
Relative mRNA level
C/ebpa
Hnf4a
Gclc
Ccn2
Col1a2
j
Cre+Veh
Cre+B022
Cre+NEC1+Z-VAD
Cre+Infliximab
Survival (%)
P=0.08
Days postinjection

Fig. S8. Hepatic neddylation deficiency promotes NIK protein expression.

a Western blot in HepG2 cells after infection with pLentiCRISPR/Cas9 lentiviruses bearing two specific human *NAE1* sgRNAs (KO1 and KO2). Cells infected with viruses expressing no sgRNA and selected with puromycin were used as a vector control (V). **b** Western blot in MLN-treated HepG2 cells at indicated concentrations for 48 h. **c** NIK (gene name: *Map3k14*) mRNA expression in mouse *Nae1*$^{f/f}$ primary hepatocytes (PH) at 48 h after infection with pAd-GFP and pAd-Cre (n = 6) and MLN-treated HepG2 cells at indicated concentrations for 48 h (n = 3). Unpaired t-tests, two-tailed. **d** Western blot of endogenous NIK in HepG2 and primary hepatocytes treated with 2.0 µM MLN for 60 h with or without 100 nM BZM in the final 12 h. **a-d**: Three biologically independent experiments. **e** Western blot and quantification in liver nuclear extracts of AAV-GFP (GFP, white bar) and AAV-Cre (Cre, red bar) mice at D21 and D24 post-virus injections (n = 3 per group). Multiple unpaired t-tests with the Holm-Sidak method. *P < 0.05; **P < 0.005. **f-g** Western blot in (**f**) liver extracts of AAV-GFP and AAV-Cre mice at D21 and D24 post-virus injections, (n = 3 per group), and (**g**) in wild-type primary hepatocytes at 48 h after infection with pAd-GFP and pAd-Cre (n = 6 per group). **h** qRT-PCR analyses in HepG2 cells treated with vehicle (Veh), 1.0 µM MLN with or without 5 µM B022 for 48 h (n = 3 per group). **i** Relative mRNA levels in livers from male D24 AAV-GFP, AAV-Cre receiving Veh or B022 mice (GFP: black, n = 6; Cre+Veh: red, n = 6; Cre+B022: blue, n = 3 in duplicates). In **(h-i)**, Two-way ANOVA followed by Tukey's multiple comparisons test. *P < 0.05; **P < 0.005; ns: not significant. **j** Male *Nae1*$^{f/f}$ mice injected with AAV-Cre received Veh (n = 7), B022 (n = 5) or combined Necrostatin 1 (NEC1)/Z-VAD-FMK (n = 5) or Infliximab (n = 8) via *i.p* injection from D10 post virus injection. Survival curves were analyzed by Log-rank (Mantel-Cox) test. All quantitative data were presented as mean ± SEM. Quantitative source data are provided as a Source Data file. Uncropped gels are provided in the Supplementary file.

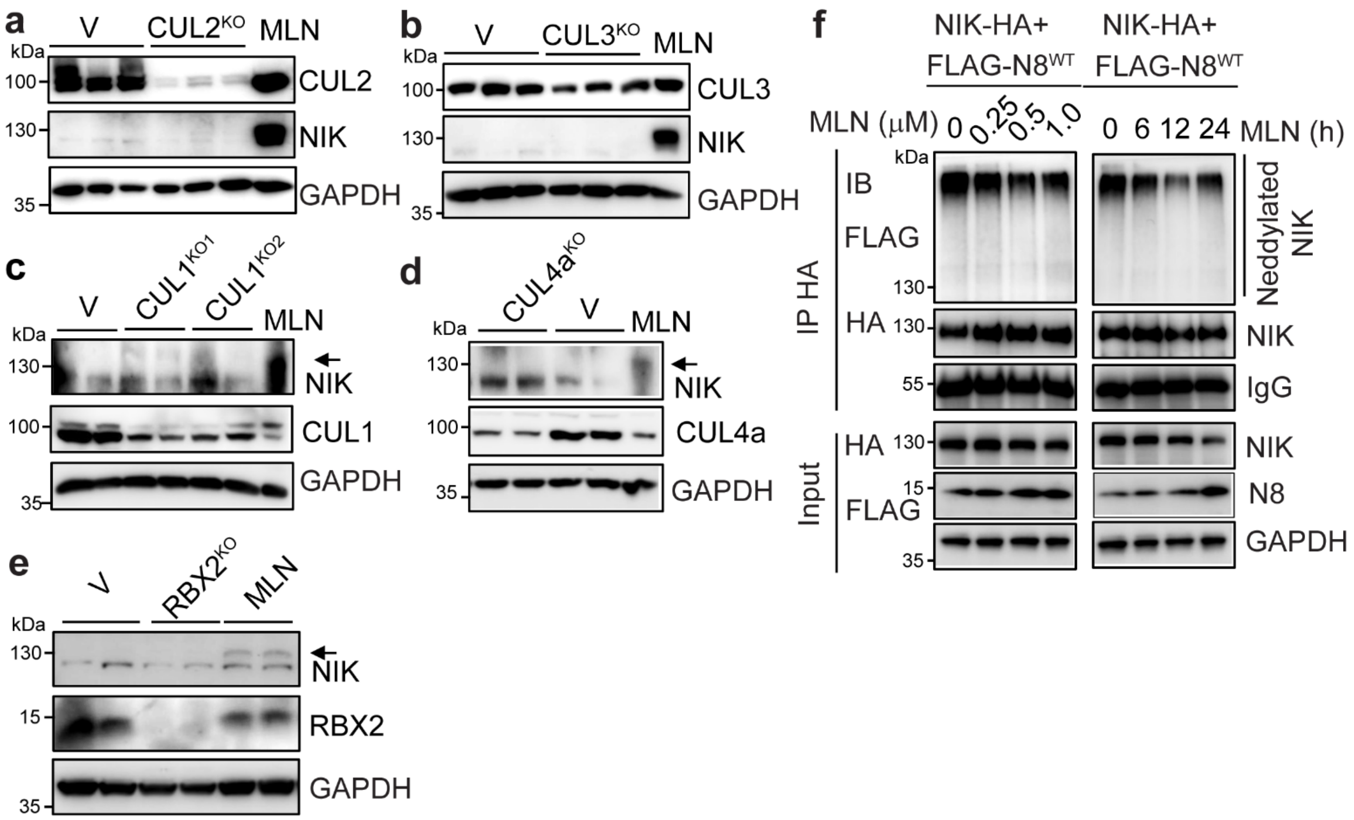

Fig. S9. Regulation of NIK by CRLs and neddylation. Western blot analyses in (**a-b**) HepG2 cells after infection with pLentiCRISPR/Cas9 lentiviruses bearing specific sgRNAs against human CUL2 ($CUL2^{KO}$) and CUL3 ($CUL3^{KO}$), respectively; and (**c-d**) NIH-3T3 cells after infection with pLentiCRISPR/Cas9 lentiviruses bearing specific sgRNAs against murine CUL1 ($CUL1^{KO1}$, $CUL1^{KO2}$) and CUL4a ($CUL4a^{KO}$), respectively; and (**e**) in RBX2 ($RBX2^{KO}$) knockout HepG2 cells targeted by CRISPR/Cas9 sgRNA. Cells infected with viruses expressing no sgRNA and selected with puromycin were used as a control (V). HepG2 cells treated with 2.0 µM MLN for 48 h were used as the control for NIK induction. **f** Twenty-four hours after transient transfection, HepG2 cells co-expressing FLAG-NEDD8 and NIK-HA were treated with indicated concentrations of MLN for 24 hours or 1.0 µM MLN for the indicated periods. The neddylation

levels of NIK were then examined by IB after IP by anti-HA magnetic beads under denaturing conditions. Three independent experiments. Uncropped gels are provided in the Supplementary file.

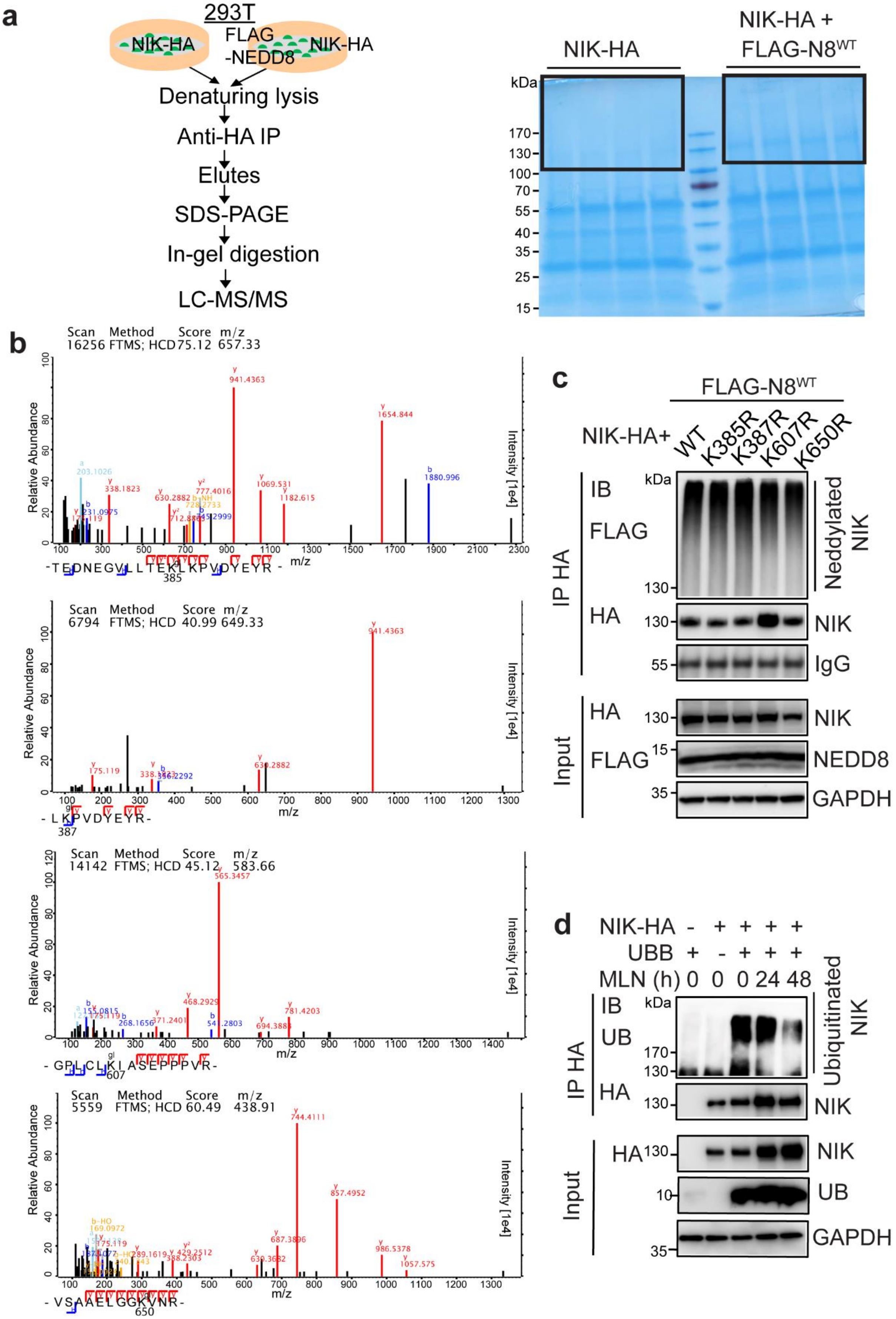
a
293T
NIK-HA
FLAG
-NEDD8
NIK-HA
Denaturing lysis
Anti-HA IP
Elutes
SDS-PAGE
In-gel digestion
LC-MS/MS
NIK-HA
NIK-HA +
FLAG-N8WT
kDa
170
130
100
70
55
40
35
25
15
b
Scan Method Score m/z
16256 FTMS; HCD 75.12 657.33
Relative Abundance
Intensity [1e4]
m/z
-TEDNEGVLLTEKLKPVDYEYR-
385
Scan Method Score m/z
6794 FTMS; HCD 40.99 649.33
-LKPVDYEYR-
387
Scan Method Score m/z
14142 FTMS; HCD 45.12 583.66
-GPLCLKIASEPPPVR-
607
Scan Method Score m/z
5559 FTMS; HCD 60.49 438.91
-VSAAELGGKVNR-
650
c
FLAG-N8WT
NIK-HA+
WT
K385R
K387R
K607R
K650R
IP HA
IB
FLAG
HA
kDa
130
130
55
Neddylated NIK
NIK
IgG
Input
HA
FLAG
130
15
35
NIK
NEDD8
GAPDH
d
NIK-HA - + + + +
UBB + - + + +
MLN (h) 0 0 0 24 48
IP HA
IB
UB
HA
kDa
170
130
130
Ubiquitinated NIK
NIK
Input
HA
130
10
35
NIK
UB
GAPDH

Fig. S10. Identification of neddylation sites. a HEK293T cells were expressed with NIK-HA alone or NIK-HA and FLAG-N8^{WT}. Forty-eight hours after transfection, NIK was immunoprecipitated by anti-HA magnetic beads under denaturing conditions. Elutes were resolved on SDS-PAGE and stained with Coomassie blue. Gels at NIK and upper were cut and subject to in-gel trypsin digestion followed by Nano LC-MS/MS. **b** Identification of K385, K387, K607, and K650 as neddylation residues. Shown were LC-MS/MS spectra of modified NIK peptides. **c** HEK293T cells were co-expressed with NIK-HA (WT) and the respective single K385R, K387R, K607R, and K650 mutants together with FLAG-N8^{WT}. Forty-eight hours after transfection, the neddylation levels of NIK and its mutants were then examined by IB after IP by anti-HA magnetic beads under denaturing conditions. **d** Twenty-four hours after transfection with the indicated constructs, HepG2 cells were treated with or without 2.0 µM MLN for indicated hours. The ubiquitination of NIK-HA was then examined by IB after IP under denaturing conditions. **c-d:** Three independent experiments. Uncropped gels are provided in the Supplementary file.

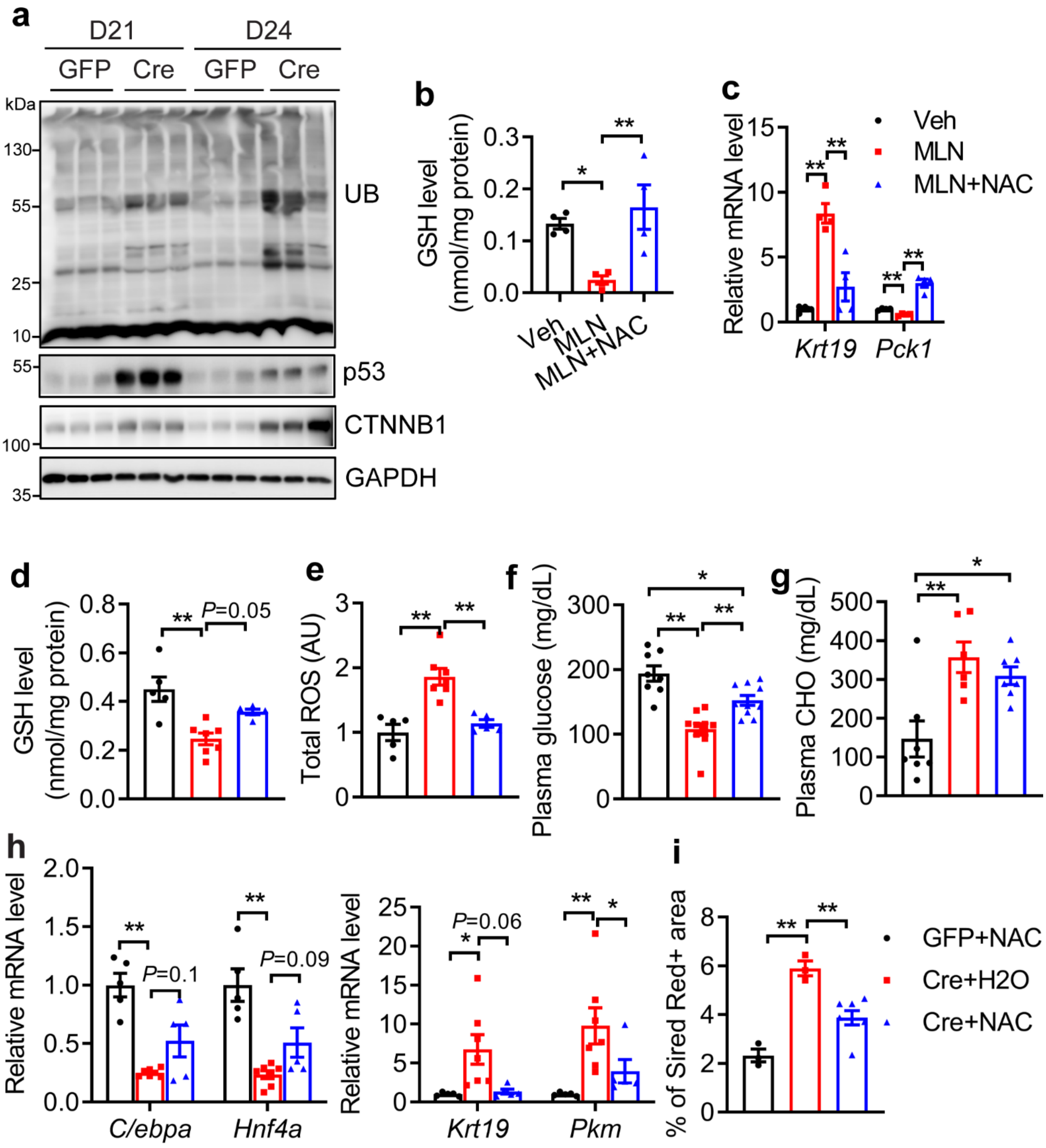

Fig. S11. NAC ameliorates NAE1 deletion-induced lethal liver failure.

a Western blot in liver extracts of AAV-GFP and AAV-Cre mice at D21 and D24 post-virus injections (n = 3 per group). **b** Total GSH levels were measured in HepG2 cells treated with vehicle (Veh), 2.0 µM MLN with or without 10 mM NAC for 48 h. Data were normalized to protein amounts (n = 4 per group). One-way ANOVA followed by Tukey's multiple comparisons test. **c**

qRT-PCR analyses in wild-type primary hepatocytes treated with Veh, 2.0 µM MLN with or without 5 mM NAC for 48 h (*n* = 4 per group). Two-way ANOVA followed by Tukey's multiple comparisons test. In (**b-c**), three independent experiments. $^*P < 0.05$, $^{**}P < 0.005$. Veh: black; MLN: red; MLN+NAC: blue. **d-i** *Nae1*$^{f/f}$ mice injected with AAV-Cre received special drinking water supplemented with 1% NAC (Cre+NAC) or regular water (Cre+H_2O) from D10 post-virus injection. AAV-GFP mice fed with the same NAC water (GFP+NAC) served as a control. **d** Liver total GSH levels were measured and normalized to protein amounts. **e** ROS levels were measured by incubating liver homogenates with a DCFH-DA probe and expressed as AU. **d-e**: GFP+NAC: *n* = 5; Cre+H2O: *n* = 7; Cre+NAC: *n* = 5. **f** Plasma glucose (GFP+NAC: *n* = 8; Cre+H2O: *n* = 10; Cre+NAC: *n* = 10) and **g** cholesterol (CHO) levels (GFP+NAC: *n* = 7; Cre+H2O: *n* = 6; Cre+NAC: *n* = 7). One-way ANOVA followed by Tukey's multiple comparisons test in (**d-g**). **h** Relative mRNA levels of indicated genes. Two-way ANOVA with Tukey's multiple comparisons test. **i** Quantification of Sirius red+ area in **Fig. 8f** (GFP+NAC: *n* = 3; Cre+H2O: *n* = 3; Cre+NAC: *n* = 7). One-way ANOVA with Tukey's multiple comparisons test. **c-i** were performed in male GFP+NAC (black), Cre+H_2O (red), and Cre+NAC (blue) mice at D24 post-virus injection. $^*P < 0.05$, $^{**}P < 0.005$. All quantitative data were presented as mean ± SEM. Quantitative source data are provided as a Source Data file. Uncropped gels are provided in the Supplementary file.

Uncropped images:

Fig. S3d. Protein expression in P10 and P14 WT and LKO livers

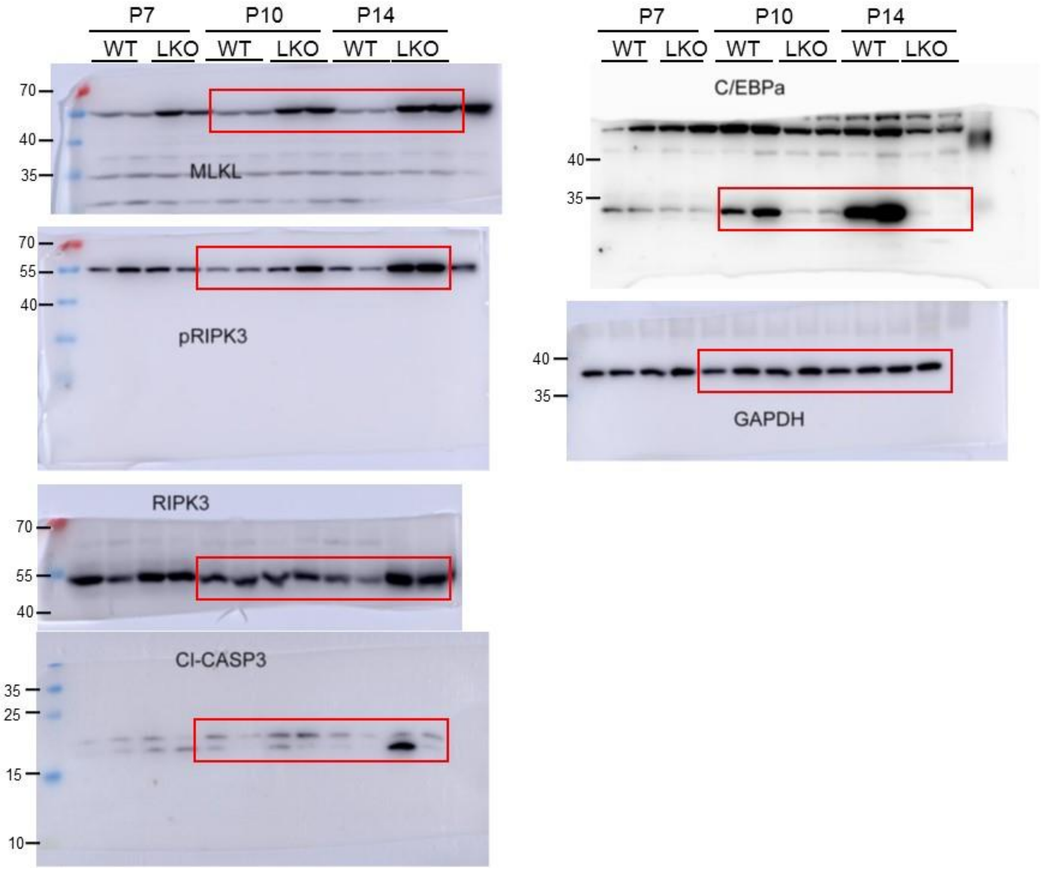

Fig. S5e. Protein expression in D14, 21 and 24 AAV-GFP and AAV-Cre livers

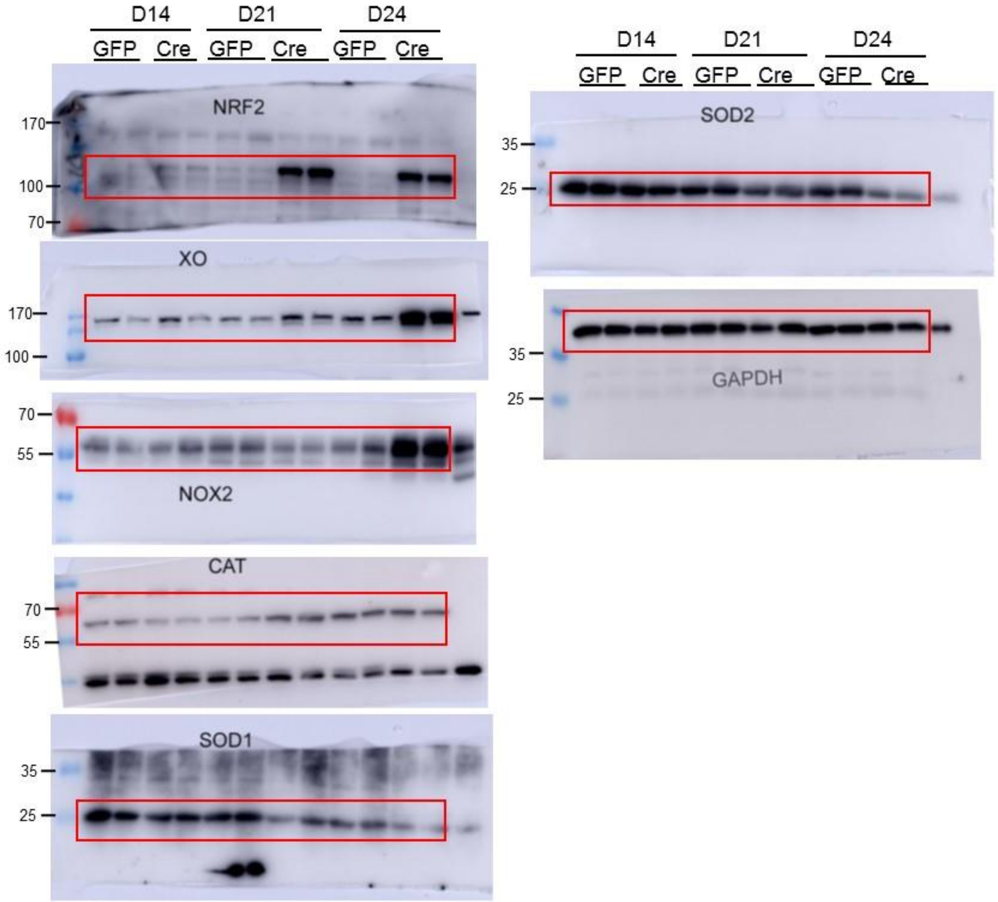

Fig. S6e Protein expression in primary hepatocytes treated with 2.0 μM MLN for 48 h with or without TNFa in the last 24 h

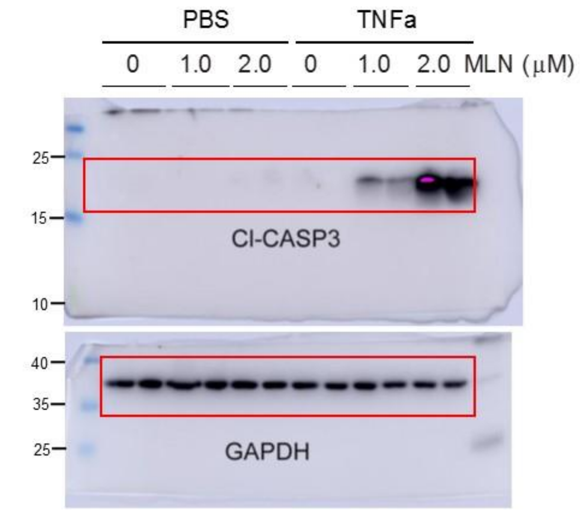

Fig. S7a. Protein expression in primary hepatocytes treated with 2.0uM MLN for 48 h

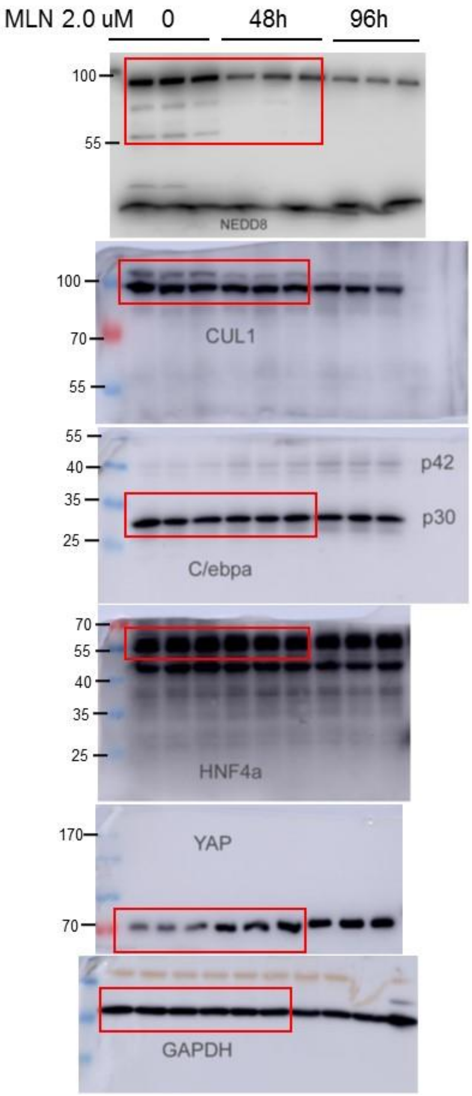

Fig. S7b. Protein expression in NAE1-deleted or MLN-treated HepG2 cells

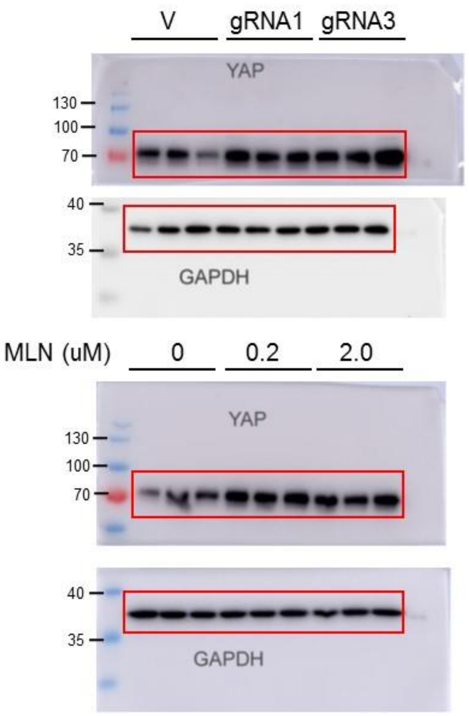

Fig. S7d. Protein expression in MLN and/or Verteporfin-treated HepG2 cells

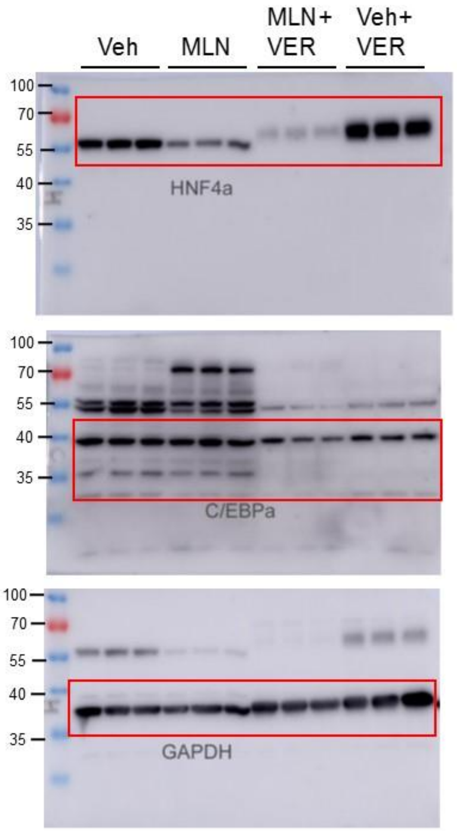

Fig. S7e. Protein expression neonatal and adult NAE1-deleted livers.

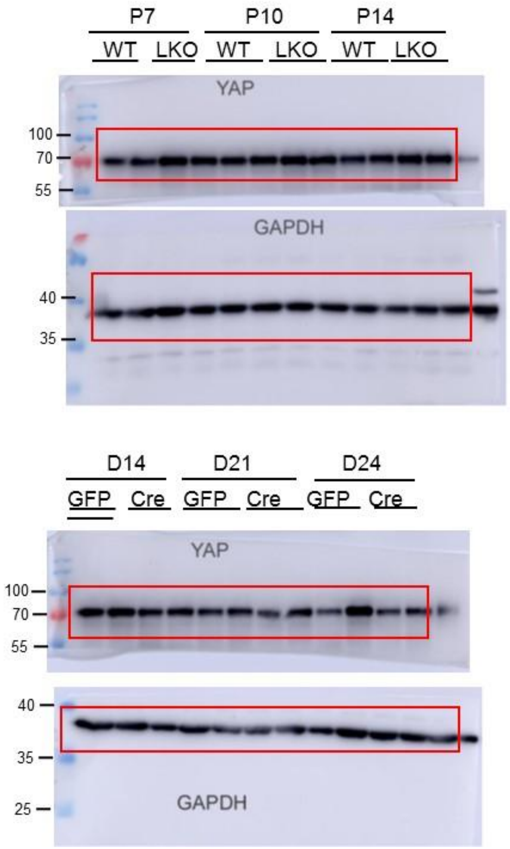

Fig. S8a Non-canonical and canonical NF-kB signaling in NAE1-deleted HepG2 cells by CRISPR/Cas9

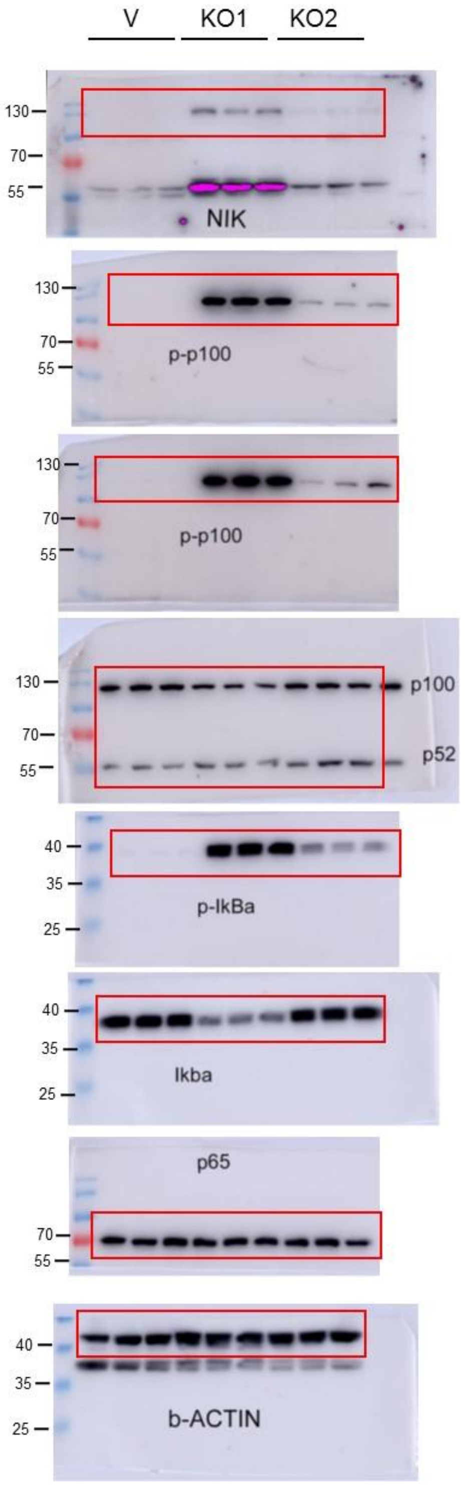

Fig. S8b protein expression in HepG2 cells treated with indicated combination of MLN and TNFa

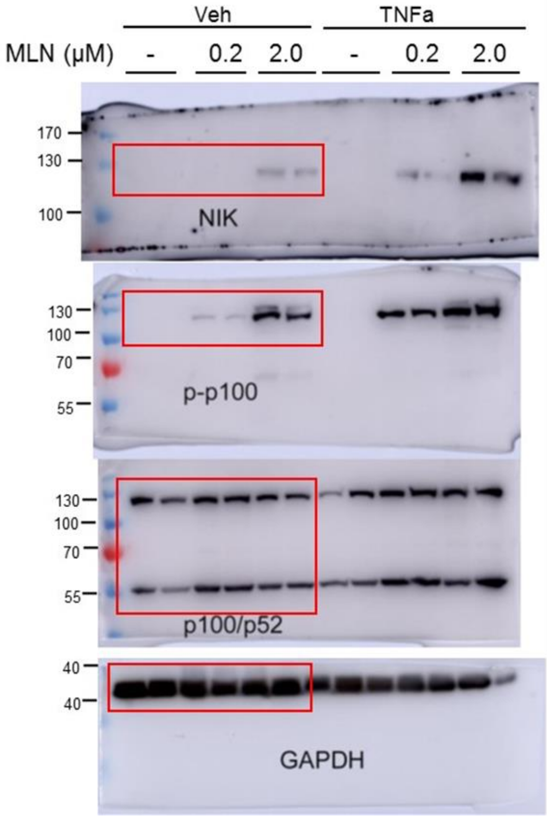

Fig. S8d Endogenous NIK expression in response to BZM and/or MLN treatment

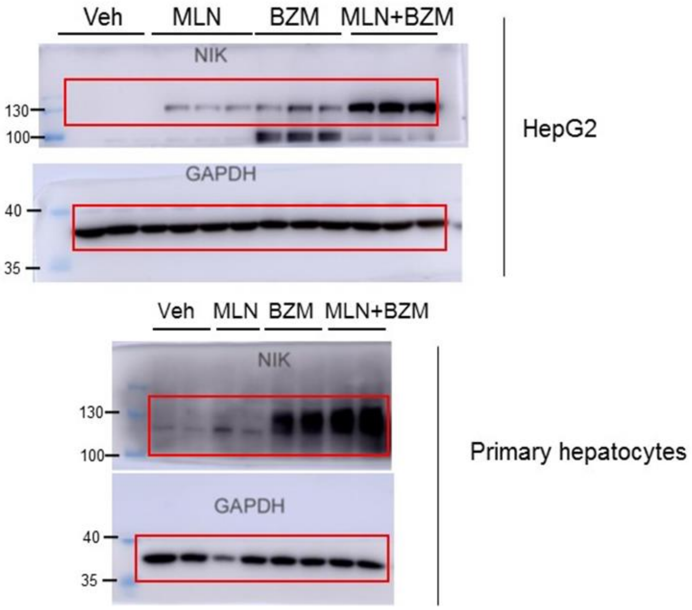

Fig. S8e protein expression in nuclear fraction of livers from D21 and D24 mice post virus injections.

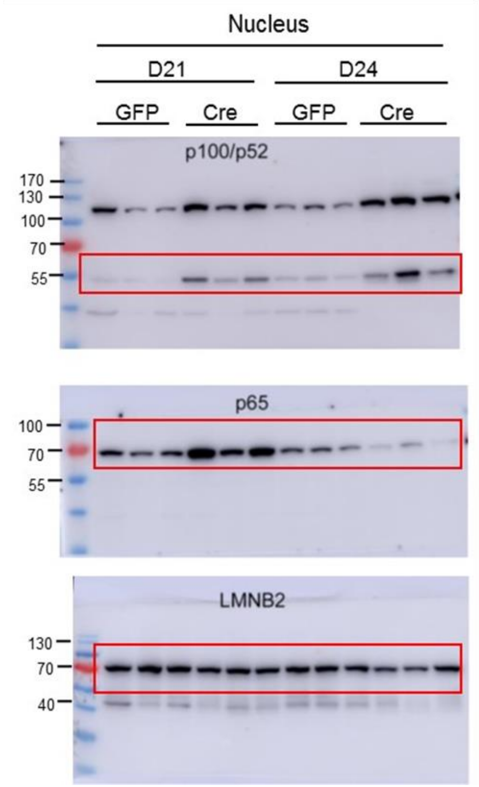

Fig. S8f Protein expression in whole liver extracts from D21 and D24 AAV-GFP and AAV-Cre mice

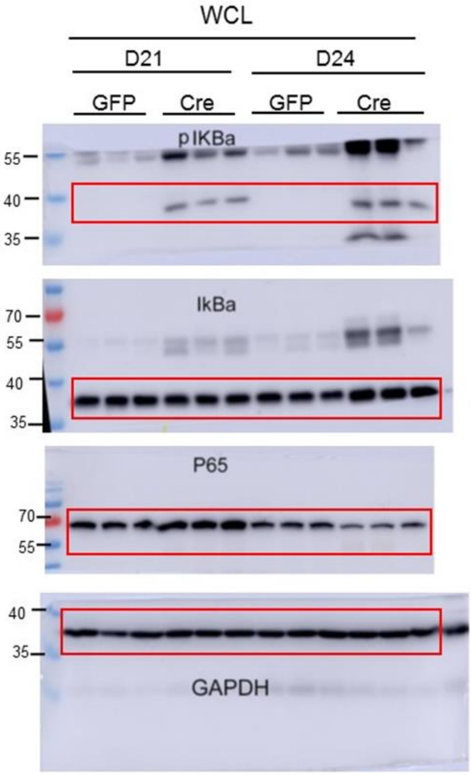

Fig. S8g Western blotting analyses of canonical NF-kB signaling in primary hepatocytes 48h after pAd-GFP or pAd-Cre infection

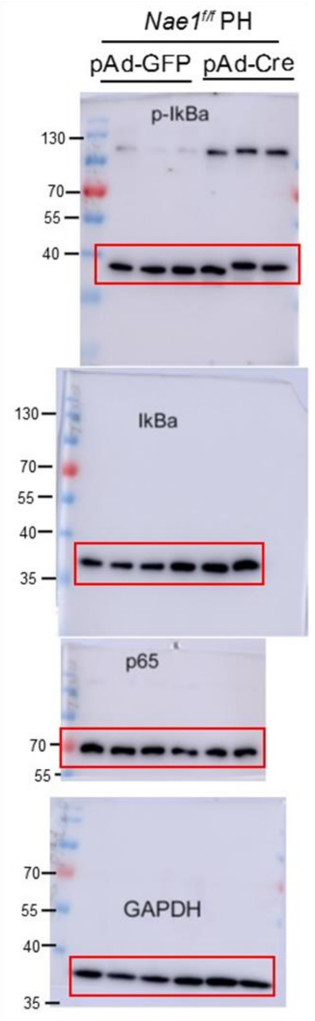

Fig. S9a-9b NIK expression in HepG2 cells with CUL2 or CUL3 deletion by CRISPR/Cas9

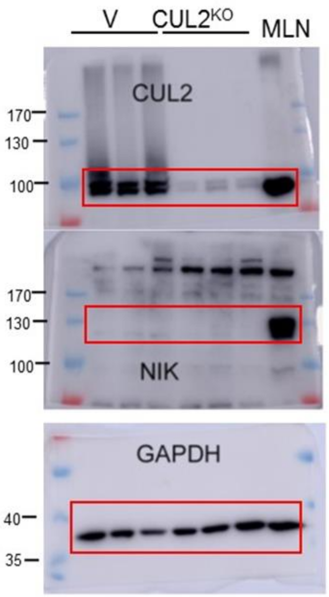

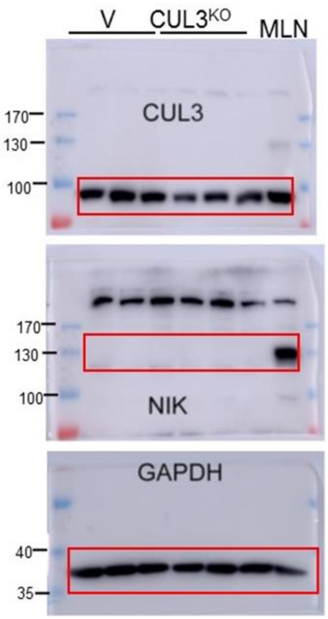

Fig S9c-9d. NIK expression in NIH-3T3 cells after CUL1 and 4a deletion by CRISPR/Cas9

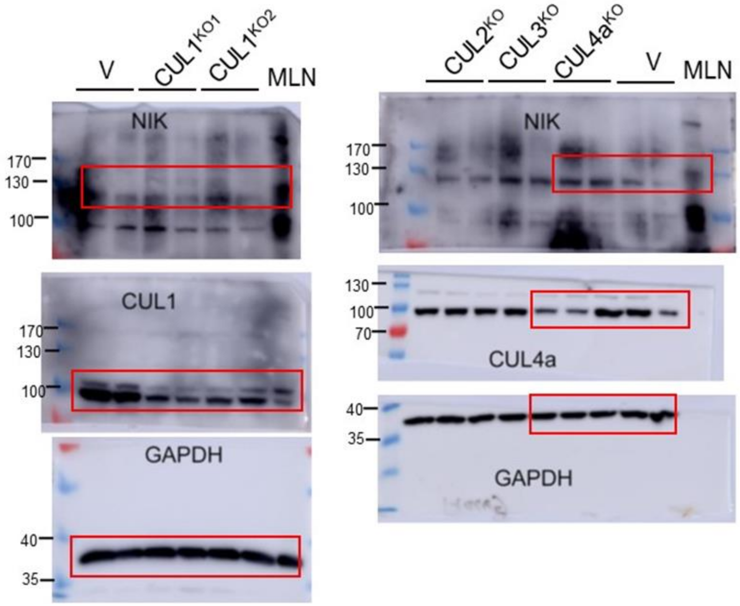

Fig S9e. NIK expression in HepG2 cells with RBX2 deleted by CRISPR/Cas9

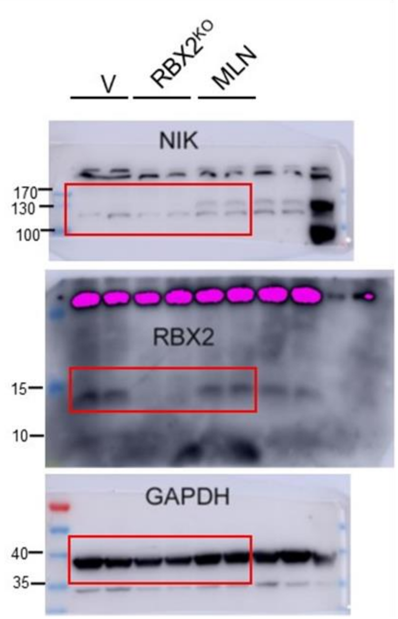

Fig. S9f Time and dose-dependent response of MLN on NIK neddylation in HepG2 cells

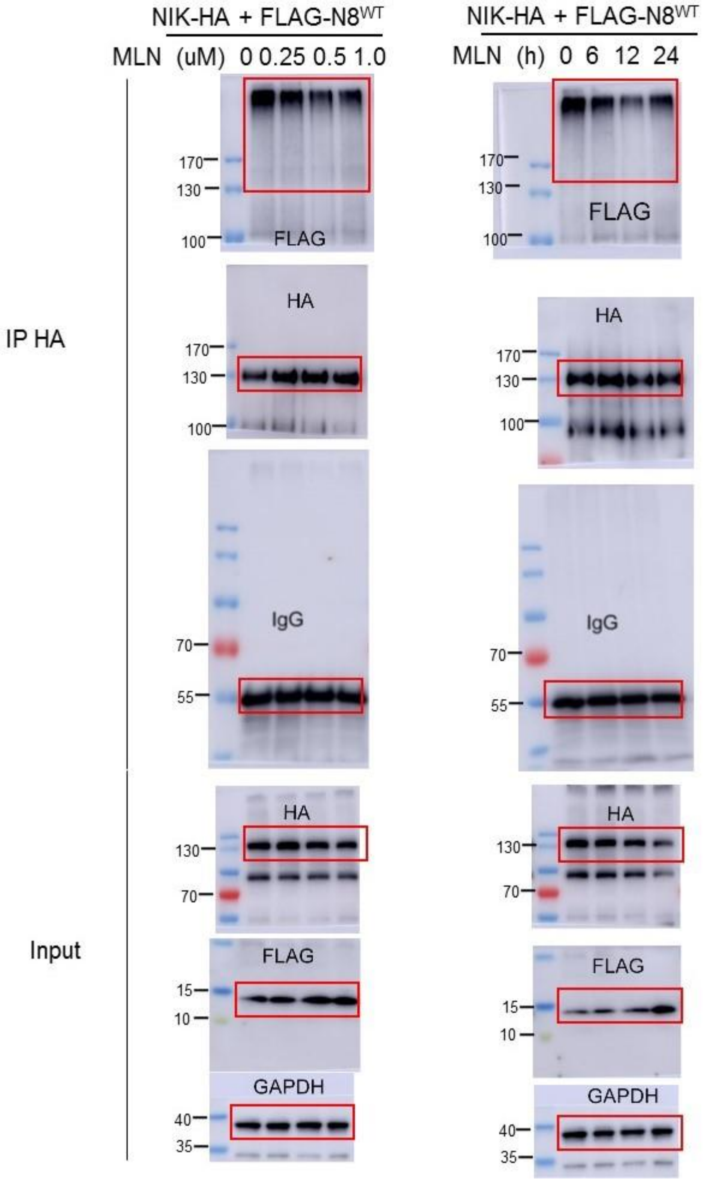

Fig. S10a SDS-PAGE and coomassie blue staining after denaturing anti-HA IP in HEK293T cells overexpressing NIK-HA alone or with FLAG-N8^{WT}

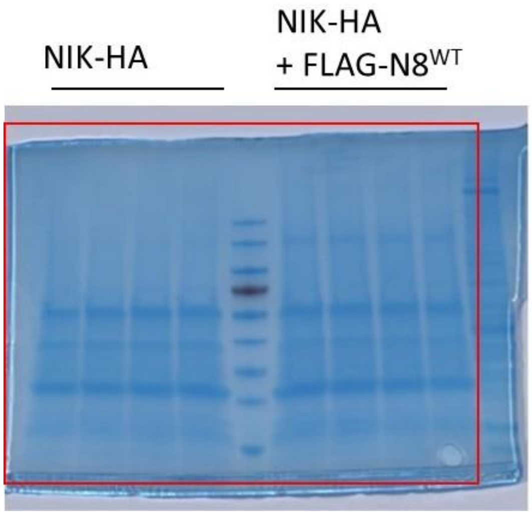

Fig. S10c Denaturing anti-HA IP in HEK293T cells overexpressing NIK-WT or various single K to R mutants together with FLAG-N8^{WT}

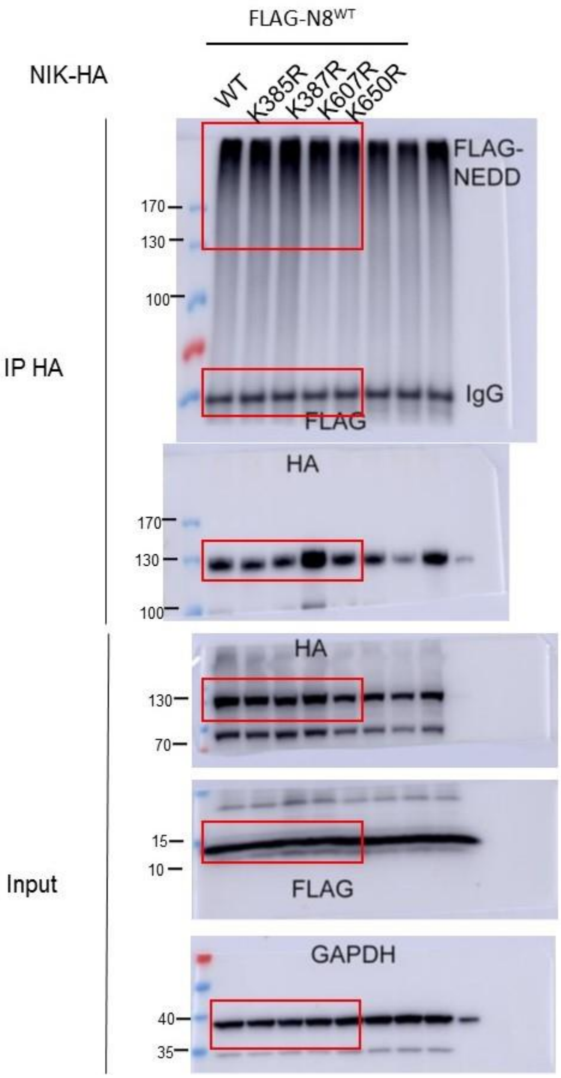

Fig. S10d Immunoprecipitation of NIK-HA in HepG2 cells overexpressed with indicated constructs

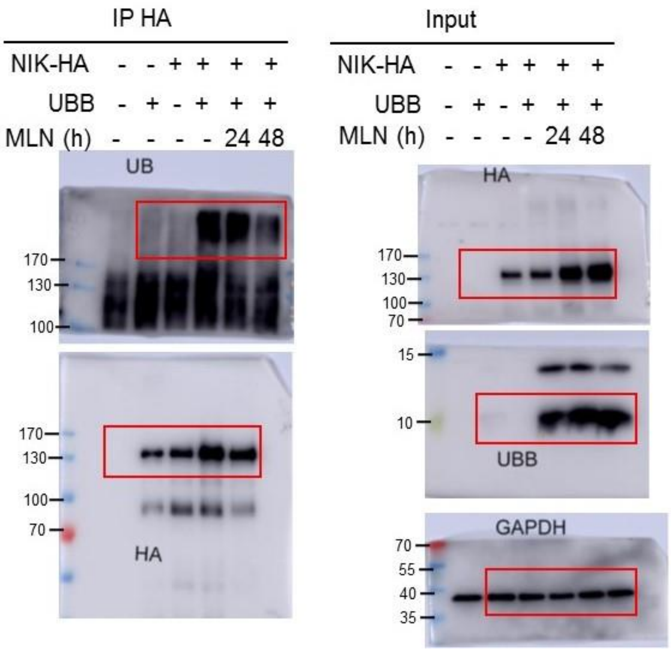

Fig. S11a Protein expression in whole liver extracts from D21 and D24 AAV-GFP and AAV-Cre mice

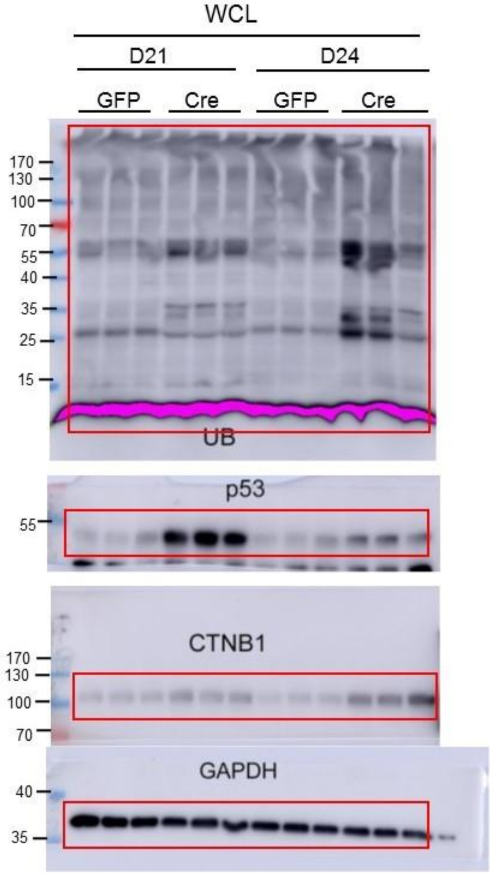

Supplementary References

1. Bligh EG, Dyer WJ. A rapid method of total lipid extraction and purification. *Can J Biochem Physiol* **37**, 911-917 (1959).

2. Nunnari JJ, Zand T, Joris I, Majno G. Quantitation of oil red O staining of the aorta in hypercholesterolemic rats. *Exp Mol Pathol* **51**, 1-8 (1989).

3. Ali SF, Lebel CP, Bondy SC. Reactive Oxygen Species Formation as a Biomarker of Methylmercury and Trimethyltin Neurotoxicity. *Neurotoxicology* **13**, 637-648 (1992).

4. Hattori M, Tugores A, Veloz L, Karin M, Brenner DA. A simplified method for the preparation of transcriptionally active liver nuclear extracts. *DNA Cell Biol* **9**, 777-781 (1990).

5. Xiao G, Sun SC. Negative regulation of the nuclear factor kappa B-inducing kinase by a cis-acting domain. *J Biol Chem* **275**, 21081-21085 (2000).

6. Lim KL, *et al.* Parkin mediates nonclassical, proteasomal-independent ubiquitination of synphilin-1: implications for Lewy body formation. *J Neurosci* **25**, 2002-2009 (2005).

7. Gao F, Cheng J, Shi T, Yeh ET. Neddylation of a breast cancer-associated protein recruits a class III histone deacetylase that represses NFkappaB-dependent transcription. *Nat Cell Biol* **8**, 1171-1177 (2006).

8. Sanjana NE, Shalem O, Zhang F. Improved vectors and genome-wide libraries for CRISPR screening. *Nat Methods* **11**, 783-784 (2014).
