# Supplementary material for: Hepatic neddylation deficiency triggers fatal liver injury via inducing NF-κB-inducing kinase in mice
Source: Nat Commun. 2022 Dec 16;13:7782. doi: 10.1038/s41467-022-35525-6 (PMC9758150; doi:10.1038/s41467-022-35525-6)
Supplement: Supplementary file 3 — Reporting Summary [file 41467_2022_35525_MOESM3_ESM.pdf]

nature portfolio

Corresponding author(s): Weiqin Chen, MS#: NCOMMS-21-06607C

Last updated by author(s): 12/07/2022

Reporting Summary

Nature Portfolio wishes to improve the reproducibility of the work that we publish. This form provides structure for consistency and transparency in reporting. For further information on Nature Portfolio policies, see our Editorial Policies and the Editorial Policy Checklist.

Statistics

For all statistical analyses, confirm that the following items are present in the figure legend, table legend, main text, or Methods section.

n/a	Confirmed	
☐	☒	The exact sample size (*n*) for each experimental group/condition, given as a discrete number and unit of measurement
☐	☒	A statement on whether measurements were taken from distinct samples or whether the same sample was measured repeatedly
☐	☒	The statistical test(s) used AND whether they are one- or two-sided<br>*Only common tests should be described solely by name; describe more complex techniques in the Methods section.*
☒	☐	A description of all covariates tested
☐	☒	A description of any assumptions or corrections, such as tests of normality and adjustment for multiple comparisons
☐	☒	A full description of the statistical parameters including central tendency (e.g. means) or other basic estimates (e.g. regression coefficient) AND variation (e.g. standard deviation) or associated estimates of uncertainty (e.g. confidence intervals)
☐	☒	For null hypothesis testing, the test statistic (e.g. *F*, *t*, *r*) with confidence intervals, effect sizes, degrees of freedom and *P* value noted<br>*Give P values as exact values whenever suitable.*
☒	☐	For Bayesian analysis, information on the choice of priors and Markov chain Monte Carlo settings
☒	☐	For hierarchical and complex designs, identification of the appropriate level for tests and full reporting of outcomes
☒	☐	Estimates of effect sizes (e.g. Cohen's *d*, Pearson's *r*), indicating how they were calculated

Our web collection on statistics for biologists contains articles on many of the points above.

Software and code

Policy information about availability of computer code

Data collection	The bcl2fastq (version 2.15.0.4) program was used to convert RNA-Seq base-calls files to fastq files. STAR version 2.0.4b was used to align the RNA-seq reads. The Cufflinks package (version 2.2.1) was used to determine DEGs.
Data analysis	Genorm algorithm (medgen.ugent.be/genorm/), the Microsoft Excel geNorm version from 2002 was used to calculate normalization factors for multiple housing keeping genes in calculating real-time PCR expression data.<br>ImageQuantTL V8.2.0 (GE Healthcare) was used to quantify Western blot.<br>Image J 1.52t was also used to quantify immunofluorescent and immunohistochemical images.<br>Statistical analysis was performed by using GraphPad Prism (Version 9.1.2, GraphPad Software, San Diego, CA, USA).<br>cor.test() function in R was used to calculate correlations.

For manuscripts utilizing custom algorithms or software that are central to the research but not yet described in published literature, software must be made available to editors and reviewers. We strongly encourage code deposition in a community repository (e.g. GitHub). See the Nature Portfolio guidelines for submitting code & software for further information.

Data

Policy information about availability of data

All manuscripts must include a data availability statement. This statement should provide the following information, where applicable:

- Accession codes, unique identifiers, or web links for publicly available datasets
- A description of any restrictions on data availability
- For clinical datasets or third party data, please ensure that the statement adheres to our policy

The RNA-Seq data generated in this study have been submitted to the SRA database with the BioProject ID: PRJNA693817 (https://www.ncbi.nlm.nih.gov/bioproject/PRJNA693817/). The previously published gene expression data from patients with hepatitis B virus-associated acute liver failure re-analyzed in this study were obtained through the Gene Expression Omnibus, GSE38941 [https://www.ncbi.nlm.nih.gov/geo/query/acc.cgi?acc=GSE38941] and GSE96851 [https://www.ncbi.nlm.nih.gov/geo/query/acc.cgi?acc=GSE96851]. The mass spectrometry proteomics data have been deposited to the ProteomeXchange Consortium via the PRIDE partner repository with the dataset identifier PXD038653. All other data generated or analyzed during this study are included in this published article (and its supplementary information files). Source data are provided in this paper.

Human research participants

Policy information about studies involving human research participants and Sex and Gender in Research.

Reporting on sex and gender	*Use the terms sex (biological attribute) and gender (shaped by social and cultural circumstances) carefully in order to avoid confusing both terms. Indicate if findings apply to only one sex or gender; describe whether sex and gender were considered in study design whether sex and/or gender was determined based on self-reporting or assigned and methods used. Provide in the source data disaggregated sex and gender data where this information has been collected, and consent has been obtained for sharing of individual-level data; provide overall numbers in this Reporting Summary. Please state if this information has not been collected. Report sex- and gender-based analyses where performed, justify reasons for lack of sex- and gender-based analysis.*
Population characteristics	*Describe the covariate-relevant population characteristics of the human research participants (e.g. age, genotypic information, past and current diagnosis and treatment categories). If you filled out the behavioural & social sciences study design questions and have nothing to add here, write "See above."*
Recruitment	*Describe how participants were recruited. Outline any potential self-selection bias or other biases that may be present and how these are likely to impact results.*
Ethics oversight	*Identify the organization(s) that approved the study protocol.*

Note that full information on the approval of the study protocol must also be provided in the manuscript.

Field-specific reporting

Please select the one below that is the best fit for your research. If you are not sure, read the appropriate sections before making your selection.

☒ Life sciences ☐ Behavioural & social sciences ☐ Ecological, evolutionary & environmental sciences

For a reference copy of the document with all sections, see nature.com/documents/nr-reporting-summary-flat.pdf

Life sciences study design

All studies must disclose on these points even when the disclosure is negative.

Sample size	Sample size of each experiment is indicated in the figure legends. Sample size was determined from preliminary experiments. Due to high cost of B022, limited sample size was used in Fig. 6i B022-treated animals (n=3 in duplicate).
Data exclusions	We excluded extreme outliers in qRT-PCR analysis based on Smirnov-Grubbs test. Data excluded was also shown in source data.
Replication	Cellular experiments were conducted at least two times independently as indicated in the Statistics and Reproducibility (Methodology section) and each legendary. All the replications tested were successful.
Randomization	Animals were randomly assigned for each drug treatment in Fig. 6 and Fig. 8. For drug-treated cell culture experiments, cells were also randomly assigned in each experiment.
Blinding	iver pathology scoring was blinded for human liver pathologist. All the gene expression experiments, biochemical analyses are also blinded during data collection before data analysis. All other experiments were not blinded due to the necessary arrangement of the sample order during data collection and analysis, but all samples were analyzed in the same way.

Reporting for specific materials, systems and methods

We require information from authors about some types of materials, experimental systems and methods used in many studies. Here, indicate whether each material, system or method listed is relevant to your study. If you are not sure if a list item applies to your research, read the appropriate section before selecting a response.

Materials & experimental systems

n/a	Involved in the study
☐	☒ Antibodies
☐	☒ Eukaryotic cell lines
☒	☐ Palaeontology and archaeology
☐	☒ Animals and other organisms
☒	☐ Clinical data
☒	☐ Dual use research of concern

Methods

n/a	Involved in the study
☒	☐ ChIP-seq
☒	☐ Flow cytometry
☒	☐ MRI-based neuroimaging

Antibodies

Antibodies used

ACTB (Mouse monoclonal, clone C4, Sigma-Millipore, MAB1501, 1/5000),
Albumin (Goat polyclonal, Novus Biologicals, NB600-41532, IF: 1/100),
Catalase (Rabbit polyclonal, GeneTex, GTX110704, 1/1000),
C/EBPα (Rabbit polyclonal, Santa Cruz, sc-61, 1/500),
CK19 (Rabbit polyclonal, Abbomax, 602-670, IF: 1/500),
Pan-Cytokeratin (Mouse monoclonal clone K4.62, Sigma Aldrich, C7159, IF: 1:20),
Cleaved caspase-3 (Rabbit polyclonal, Cell signaling technology, 9661, 1/1000),
Cleaved caspase-8 (Rabbit polyclonal, Cell signaling technology, 9429, 1/1000),
CTNB1 (Rabbit monoclonal, clone D10A8, Cell signaling technology, 8480, 1/1000),
CUL1 (Rabbit polyclonal, Santa Cruz sc-11384, 1/1000),
CUL2 (Rabbit monoclonal, clone EPR3104(2), Abcam, ab166917, 1/1000),
CUL3 (Rabbit polyclonal, Cell signaling technology, 2759, 1/1000),
CUL4a (Rabbit polyclonal, Novus Biologicals, NB100-2267, 1/1000)
Desmin (Mouse monoclonal, clone DE-U-10, Sigma Aldrich, D1033, IF: 1:40),
FLAG (Mouse monoclonal, clone M2, Sigma Aldrich, F1804, 1/1000),
F4/80 (Rat monoclonal, clone Cl:A3-1, Bio-Rad, MCA497GA, IF: 1:50),
GAPDH (Mouse monoclonal, clone 1E6D9, Proteintech, 60004-1-IG, 1/20000),
GFP (Rabbit polyclonal, Abcam, ab290, 1/1000),
HA (Mouse monoclonal, clone C29F4, Cell signaling technology, 3724, 1/1000),
HNF4α (Rabbit monoclonal, clone F.674.9, Thermo Fisher Scientific, MA5-14891, IF: 1/500),
HNF4α (Mouse monoclonal, clone K9218, Thermo Fisher Scientific, MA1-199, 1/1000),
IκBα (Mouse monoclonal, clone L35A5, Cell signaling technology, 4814, 1/1000),
p-IκBα (Rabbit monoclonal, clone 14D4, Cell signaling technology, 2859, 1/1000),
Ki-67 (Rabbit monoclonal, clone D3B5, Cell signaling technology, 9129, IF: 1/400),
LMNB2 (Rabbit polyclonal, Proteintech, 10895-1-AP, 1/2000),
MLKL (Rabbit polyclonal, Abcam, ab172868, 1/1000),
Myc (Mouse monoclonal, clone 9B11 Cell signaling technology, 2276, 1/1000),
NAE1 (Rabbit monoclonal, clone D9I4Z, Cell signaling technology, 14321, 1/1000),
NEDD8 (Rabbit monoclonal, clone 19E3, Cell signaling technology, 2754, IB: 1/1000, IF: 1/300),
p-NF-κB2 p100 (Rabbit polyclonal, Cell signaling technology, 4810, 1/1000),
NF-κB2 p100/p52 (Rabbit polyclonal, Cell signaling technology, 4882, 1/1000),
NIK (Rabbit polyclonal, Cell signaling technology, 4994, 1/1000),
NIK (Mouse monoclonal, clone A-12, Santa Cruz Biotechnology sc-8417, IP: 1 μg per 500 μg of total protein),
NOX2 (Mouse monoclonal, clone 53, BD Biosciences, 611414, 1/1000),
NRF2 (Rabbit monoclonal, clone D1Z9C, Cell signaling technology, 12721, 1/1000),
p53 (Rabbit monoclonal, clone D2H9O, Cell signaling technology, 32532, 1/500),
p65 (Rabbit monoclonal, clone D14E12, Cell signaling technology, 8242, 1/1000),
p-RIPK3(T231 + S232) (Rabbit monoclonal, clone EPR19403-52, Abcam, ab201912, 1/5000),
RBX2 (Rabbit polyclonal, Proteintech 11905-1-AP, 1/1000),
RIPK3 (Rabbit polyclonal, Novus biologicals, NBP1-77299, IB: 1/2000, IHC:1/200),
SOD1 (Rabbit polyclonal, Proteintech, 10269-1-AP, 1/1000),
SOD2 (Rabbit polyclonal, Proteintech, 24127-1-AP, 1/5000),
UBC12 (Rabbit monoclonal, clone D13D7, Cell signaling technology, 5641, 1/1000),
UBC12 (Mouse monoclonal, clone D-4, Santa Cruz, sc-390064, IP: 1 μg per 500 μg of total protein),
Ubiquitin (Mouse monoclonal, clone P4D1, Cell signaling technology, 3936, 1/1000),
Xanthine oxidase (Mouse monoclonal, clone A-3, Santa Cruz, sc-398548, 1/500),
YAP (Rabbit monoclonal, clone D8H1X, Cell signaling technology, 14074, 1/1000)

We used the following secondary antibodies:
Alexa Fluor 488-conjugated goat anti-Rabbit IgG (H+L) (ThermoFisher, A11034, 1/500),
Alexa Fluor 594-conjugated F(ab')2-Goat anti-Rabbit IgG (H+L) (ThermoFisher, A11072, 1/500),

	Alexa Fluor Plus 594-conjugated donkey anti-Goat IgG (H+L) (ThermoFisher, A32758, 1/500),<br>Alexa Fluor 488-conjugated donkey anti-rabbit IgG (H+L) (ThermoFisher, A21206, 1/500),<br>Alexa Fluor 594-conjugated goat anti-mouse IgG (H+L) (ThermoFisher, A11032, 1/500),<br>Alexa Fluor 647, Goat anti-Rat IgG (H+L) Cross Adsorbed Secondary Antibody (ThermoFisher, A21247, 1/500)<br>HRP-conjugated goat anti-rabbit IgG (H+L) (Bio-rad, 1706515, 1/300)<br>HRP-conjugated goat anti-mouse IgG (H+L) (Bio-rad, 1706516, 1/300)
Validation	Antibodies were chosen for specific applications based on suppliers' recommendations indicated on their websites and all showed bands of expected molecular weight. All antibodies have been cited by other research publications.<br>Antibodies to HNF4a (MA1-199) was verified by knockdown to ensure that the antibody binds to the antigen stated. Antibody to HNF4a (MA5-14891) was verified by relative expression to ensure that the antibody binds to the antigen stated. Antibodies to LMNB2, SOD1 were validated by KO/KD.

Eukaryotic cell lines

Policy information about cell lines and Sex and Gender in Research

Cell line source(s)	HepG2: ATCC HB-8065; HEK-293: ATCC CRL-1573; 293T: ATCC CRL-3216; NIH-3T3: CRL-1658
Authentication	All the cell lines were authenticated by ATCC. For HepG2, HEK-293 and 293T, STR profiling was used for authentication by ATCC.
Mycoplasma contamination	All the cell lines were tested by ATCC with no mycoplasma contamination
Commonly misidentified lines<br>(See ICLAC register)	No commonly misidentified lines were used in this manuscript.

Animals and other research organisms

Policy information about studies involving animals; ARRIVE guidelines recommended for reporting animal research, and Sex and Gender in Research

Laboratory animals	Male and female mice ranging from postnatal 7 days up to 5 months old were used in this study.<br>Nae1 floxed mice, SENP8 WT and SENP8 KO mice were obtained from Dr. Huabo Su at Augusta University. Albumin-Cre mice (JAX#: 003574) and C57BL/6J mice (JAX#000664) were obtained from Jackson Laboratory. Animals were kept under a controlled temperature around 21C and 50% humidity with a 12h/12h light and dark cycle.
Wild animals	No wild animals were used in this study.
Reporting on sex	We specifically presented data showing sexual dimorphism in mice with embryonic-onset hepatocyte-specific deletion of NAE1 (Fig. 1 and Fig. S1 as well as Source Data). Due to the expensive cost of the AAV viruses as well as the experimental drugs, such as B022, we did not test whether deletion of NAE1 in hepatocytes of adult female mice renders similar phenotype, and whether B022 or NAC are effective in alleviating phenotype in female mice.
Field-collected samples	No field-collected samples were used in this study.
Ethics oversight	All animal experiments were done according to the NIH guidelines for the care and use of laboratory animals, and approved by the IACUC at Augusta University (approve #: 2012-0462).

Note that full information on the approval of the study protocol must also be provided in the manuscript.
